# Projected Future Habitat Suitability of Five Endangered Terrestrial Mammals in South Korea Under CMIP6 Climate Scenarios

**DOI:** 10.3390/ani16162489

**Published:** 2026-08-10

**Authors:** Gyeong-Min Lee, Yeong-Seok Jo

**Affiliations:** Department of Biology Education, Daegu University, Gyeongsan 38453, Republic of Korea; lgm0109@naver.com

**Keywords:** MaxEnt, species distribution models, CMIP6, *Lutra lutra*, *Martes flavigula*, *Prionailurus bengalensis*, *Naemorhedus caudatus*, *Pteromys volans*

## Abstract

Global climate change threatens regional biodiversity, yet multi-model assessments tracking future range shifts of endangered mammals remain limited. This study evaluated future habitat suitability for five legally protected terrestrial mammals in the Republic of Korea (*Lutra lutra*, *Martes flavigula*, *Prionailurus bengalensis*, *Naemorhedus caudatus*, and *Pteromys volans*). Using the MaxEnt framework, we projected distributions across four Shared Socioeconomic Pathways (SSPs) and future horizons (2021–2100) using five CMIP6 global climate models (GCMs). Projections revealed distinct, species-specific dynamics across consensus models. Four taxa (*L. lutra*, *P. bengalensis*, *N. caudatus*, and *P. volans*) exhibited progressive range contractions, with suitable habitats declining by −95% to −100% under high carbon emission scenarios. Conversely, *M. flavigula* demonstrated expanding trajectories, reflecting high-latitude range-edge responses. Prominent inter-model variations in the INM-CM5-0 and BCC-CSM2-MR models highlighted predictive uncertainties. We conclude that real-world habitat occupancy will be heavily constrained by non-climatic anthropogenic barriers, including fence networks, road systems, and climate-driven extensive wildfires. Consequently, proactive conservation should prioritize reinforcing verified contemporary core habitats along the Baekdudaegan Mountain Range and establishing structural corridors rather than relying solely on volatile bioclimatic projections.

## Simple Summary

Climate change severely threatens wildlife, but we often lack comprehensive regional predictions for how endangered animals will move. This study investigated how future climate change will affect the habitats of five legally protected mammals in the Republic of Korea: the Eurasian otter, yellow-throated marten, leopard cat, long-tailed goral, and Siberian flying squirrel. Using advanced computer modeling and multiple global climate change projections up to the year 2100, we mapped areas that will remain suitable for these species. Our results showed that four out of the five species will face drastic habitat loss, losing up to 100% of their suitable areas under high-greenhouse-gas-emission scenarios, indicating high vulnerability to regional climate shifts. In contrast, the yellow-throated marten showed potential for expansion. However, real-world survival will be heavily restricted by human activities, including road networks, wildlife fences, and extensive forest fires. We conclude that active conservation efforts must move beyond simple climate projections. Instead, society should prioritize protecting verified current habitats and establishing connected wildlife corridors. This research provides a crucial baseline for policymakers to design effective wildlife protection plans and sustainable infrastructure to secure a biodiversity-safe future.

## 1. Introduction

Global climate change is an overriding environmental challenge that induces profound shifts in the structure, function, and stability of terrestrial ecosystems worldwide [1,2]. Alterations in temperature regimes and precipitation patterns, along with the increasing frequency of extreme weather events, directly jeopardize biodiversity by exceeding the physiological tolerances of species and driving widespread habitat fragmentation [3,4]. These anthropogenic climatic shifts do not merely impact individual species in isolation. Rather, they trigger trophic cascading repercussions throughout food webs, ultimately degrading the delivery of critical ecosystem services [5,6].

Within trophic cascades, apex predators and keystone species play an indispensable role in maintaining biological communities and regulating top-down ecological processes [7]. Because these species possess expansive home ranges and specific microhabitat requirements, they are exceptionally vulnerable to climate-induced habitat change and environmental disturbances [8]. In fragmented landscapes, the contraction of suitable habitats for keystone species alters their spatial distribution and marking behaviors, which are fundamental to their population dynamics and territorial socio-ecology [9]. Consequently, tracking and predicting the range shifts of these ecological indicators serves as a robust proxy for evaluating broader ecosystem health and landscape-level resilience under future climate scenarios.

Recently, beyond a simple descriptive analysis of current species distributions, future predictive analysis reflecting climate change scenarios has emerged as a core tool in endangered species research [10]. Species Distribution Models (SDMs) can predict potential patterns of distributional changes by combining current species occurrence data with environmental variables to estimate habitat suitability, and subsequently applying these parameters to future climate conditions [11,12]. The importance of this modeling approach is being increasingly emphasized, particularly as it can be effectively utilized for delineating protection priority areas, establishing long-term conservation strategies, and implementing preemptive management for species vulnerable to climate change [13].

The Korean Peninsula presents a unique biogeographical crucible for examining these climate-driven dynamics. The Korean Peninsula is characterized by complex mountainous topography and bounded by distinct marine environments. These geographical features create pronounced climatic gradients that subject native mammals to the ‘peninsula effect’, exacerbating genetic isolation and restricting northward range shifts under climate warming [14]. Despite this ecological urgency, macroecological assessments that bridge the gap between SDMs and the spatial quantification of ecosystem services remain highly limited in East Asia. Most existing frameworks utilize foundational bioclimatic models without integrating the precise ecological parameters of native indicators or evaluating how range shifts compromise localized ecological functions.

Furthermore, rather than simply presenting whether future distributions will change, a robust approach must consider the inherent uncertainty within prediction results to establish reliable conservation strategies in response to climate change [13,15]. In particular, when space-based decision making is required, such as designating protected areas or determining management priorities, predictions based on a single climate scenario cannot guarantee long-term conservation effectiveness [16,17]. In this regard, future habitat prediction applying the CMIP6-based Shared Socioeconomic Pathways (SSPs) is drawing considerable attention as an analytical framework that can more comprehensively evaluate the potential response range of species to climate change by simultaneously considering various greenhouse gas emission pathways and multiple climate models [16,17]. Studies predicting species distributions utilizing the CMIP6–SSP framework have recently been increasing as well, contributing to the expansion of empirical and scientific evidence necessary for establishing climate change-responsive wildlife management and conservation policies [18,19].

To address these critical knowledge gaps, this study utilizes the maximum entropy (MaxEnt) algorithm integrated with the latest Coupled Model Intercomparison Project Phase 6 (CMIP6) climate projections to simulate the spatial redistributions of key mammalian indicators across the Korean Peninsula. By incorporating robust, long-term field survey data and evaluating multi-faceted environmental covariates, we aim to: (1) delineate the current and future bioclimatic velocity and habitat suitability for target keystone species; (2) quantify the spatial variance and vulnerability of ecosystem services associated with these range shifts; and (3) establish empirically grounded, proactive spatial priorities for wildlife conservation and climate mitigation policy. Our findings provide an essential macroecological framework for safeguarding biodiversity corridors and sustaining ecosystem integrity under accelerating global change.

## 2. Materials and Methods

### 2.1. Study Area

Republic of Korea is located between approximately 33°06′ N and 38°36′ N latitude and 125°04′ E and 131°52′ E longitude, and due to its topographical characteristics, approximately 70% of the national territory consists of forests [18]. Although it belongs to the temperate climate zone with four distinct seasons, the regional climate varies widely owing to the elongated topography from north to south and the influences of continental and coastal air currents. The annual mean temperature is generally distributed within the range of 6–16 °C, and the annual mean precipitation is approximately 1200 mm, of which more than half (50–60%) is concentrated between June and August, which includes the summer monsoon period [9]. Vegetation exhibits distribution patterns according to temperature zones and is classified into five zones: warm temperate forest, cool temperate forest-southern, cool temperate forest-central, cool temperate forest-northern, and subarctic forest zone [20].

### 2.2. Modeling Methods

#### 2.2.1. Occurrence Data

Currently, 20 mammal species are designated as Endangered by the Ministry of Environment in the Republic of Korea. To select robust target taxa for species distribution modeling, we applied a systematic three-step screening protocol. First, three marine mammal species (*Callorhinus ursinus*, *Eumetopias jubatus*, and *Phoca largha*) were excluded due to their distinct non-terrestrial spatial characteristics. Second, seven species that are already extirpated from the wild in the Republic of Korea (e.g., *Ursus thibetanus*, *Panthera pardus*, *P. tigris*, *Lynx lynx*, *Vulpes vulpes*, *Canis lupus*, and *Cervus nippon*) were omitted. Third, five extremely rare species (*Murina ussuriensis*, *Plecotus ognevi*, *Myotis rufoniger*, *Mustela nivalis*, and *Moschus moschiferus*) were excluded because their limited presence records were insufficient to achieve acceptable model performance (yielding low Area Under the Curve values, AUC ~ 0.5) [21,22]. Consequently, we used spatial occurrence data of the remaining five terrestrial species of endangered mammals (*Lutra lutra*, *Martes flavigula*, *Prionailurus bengalensis*, *Naemorhedus caudatus*, and *Pteromys volans*).

The occurrence data used in this study were compiled and organized based on nationwide wildlife survey datasets conducted by MCEE and its affiliated institutions. Spatial coordinates of species presence were extracted from the nationwide Wildlife Survey and the Endangered Species Survey managed by the National Institute of Biological Resources (NIBR, Incheon, Republic of Korea), as well as the 3rd (2006–2010), 4th (2011–2015), and 5th (2016–2020) National Natural Environment Surveys conducted by the National Institute of Ecology (NIE, Seocheon, Republic of Korea). These surveys represent standard nationwide biodiversity datasets systematically executed across the country since 2006 [9,18].

The presence records encompassed diverse field methodologies, including sign tracking, direct observation, camera trapping, and roadkill data. To ensure spatial accuracy and ecological validity, data verification was implemented following the procedures described by Jo et al. [9]. First, presence coordinates were visualized using the ‘Add XY Data’ function in ArcGIS v10.6 (ESRI, Redlands, CA, USA) to identify locations that were explicitly inconsistent with the ecological characteristics of the species or that indicated potential recording errors. Subsequently, supplementary information, including GPS metadata, field photographs, and original survey reports, was thoroughly reviewed, and records with low spatial reliability were excluded from the analysis.

To account for spatial autocorrelation and sampling bias, occurrence records were filtered based on species-specific home range and mobility criteria. A total of 29,867 records for *Lutra lutra*, 26,711 for *Martes flavigula*, 51,136 for *Prionailurus bengalensis*, 4626 for *Naemorhedus caudatus*, and 31,137 for *Pteromys volans* were retained for model training after spatial filtering (Table S1).

#### 2.2.2. Environmental Variables and Climate Change Scenarios

To evaluate future habitat suitability, bioclimatic variables were downloaded from the WorldClim v2.1 database (https://www.worldclim.org). Raster datasets with a spatial resolution of 30 arc-seconds (~1 km at the latitude of the study area) were collected. The environmental datasets comprised 19 bioclimatic variables (BIO1–BIO19) and a Digital Elevation Model (DEM) obtained from the Environmental Geographic Information Service (EGIS; https://egis.me.go.kr) maintained by the MCEE (Table 1). The structure and selection of these environmental covariates were adapted based on previously validated mammalian distribution models in the Republic of Korea [18,23].

Future habitat suitability was projected using the Coupled Model Intercomparison Project Phase 6 (CMIP6) climate framework. CMIP6 serves as an international climate modeling framework designed to compare various Global Climate Models (GCMs) under unified experimental designs and socioeconomic scenario structures, and it is standardly utilized in research evaluating the impacts of climate change on ecosystems and biodiversity [16,17].

Future climatic conditions were analyzed across four distinct time periods (2021–2040, 2041–2060, 2061–2080, and 2081–2100), based on the SSPs. For each period, 4 SSP scenarios were applied (SSP1–2.6, SSP2–4.5, SSP3–7.0, and SSP5–8.5). Furthermore, to accurately capture the specific climatic characteristics of the Korean Peninsula, 5 GCMs were selected and integrated into the analysis. The 5 GCMs included: GISS-E2-1-G developed by the NASA Goddard Institute for Space Studies (USA) [24,25]; ACCESS-CM2 by CSIRO (Australia) [26]; INM-CM5-0 by the INM (Russia) [27]; BCC-CSM2-MR by the NCC (China) [28]; and MRI-ESM2-0 jointly developed by JAMSTEC and the MRI (Japan) [29]. These climate models have been reported to effectively reflect climate variability in East Asia [19].

By combining the 5 GCMs, 4 SSP scenarios, and 4 time periods, the inherent uncertainties within future climate projections were systematically incorporated. This multi-model, multi-scenario ensemble approach accounts for the predictive uncertainties of future climate changes and contributes to a more robust and stable assessment of potential species-specific habitat shifts [13,15].

The environmental variables were preprocessed using the WGS84 (EPSG:4326) geographic coordinate system. To ensure spatial consistency across the analysis, all environmental layers were clipped to the terrestrial extent of the Republic of Korea using the ‘Extract by Mask’ tool in ArcGIS, based on the administrative boundaries provided by GADM v2.5 (gadm.org). Subsequently, all raster layers were standardized to share identical spatial extents, cell sizes, and alignment. To maintain compatibility with the MaxEnt modeling platform, the environmental datasets were converted from raster format to ASCII (.asc) format using the ‘Raster to ASCII’ tool in ArcGIS.

#### 2.2.3. Species Distribution Modeling

Species distribution projections were performed using MaxEnt v3.4.4 (American Museum of Natural History, New York, NY, USA), strictly adhering to the standard modeling workflows described by Young et al. [30]. Occurrence datasets were formatted into CSV files containing species names, latitudes, and longitudes. To mitigate spatial sampling bias caused by duplicate presence points at identical locations, the ‘Remove duplicate presence records’ option was activated [11]. To prevent autocorrelation, close occurrences within a radius of each species’ home range were deleted using the ‘Near’ function in ArcGIS.

For model training and validation, the ‘Random seed’ option was enabled, randomly partitioning 25% of the total presence data as an independent test dataset [12,31]. To evaluate model stability and quantify variance among the projections, the number of replicates was set to 15 using the subsampling technique [32].

To prevent model overfitting, the regularization multiplier was maintained at its default value of 1.0 [33]. The feature class settings were configured to ‘Auto features’, allowing the automatic selection of linear, quadratic, and hinge features based on the sample size of the input data [12]. We adopted default parameter settings to balance model complexity and predictive performance effectively. This standardized approach prevents model overfitting across broad geographic scales without introducing species-specific tuning biases [31]. The maximum number of iterations was set to 5000 with a convergence threshold of 0.00001 to ensure optimal model convergence [32].

The number of background points was established at the default value of 10,000 [22]. A spatial bias file was not implemented because the occurrence datasets were derived from official national survey databases maintained by NIE and NIBR. These institutional datasets undergo systematic quality assurance and quality control (QA/QC) protocols, including expert validation of spatial accuracy and sampling effort across national grid units, inherently minimizing spatial sampling bias prior to model calibration. The model output was configured to the logistic format [11]. Model performance was evaluated using the Area Under the Receiver Operating Characteristic Curve (AUC), fractional omission rate at the 10th percentile training presence threshold, and True Skill Statistic (TSS) (Table S2). Additionally, to account for instances where future environmental conditions might exceed the range of the baseline climate data, the ‘Extrapolate’ and ‘Clamping’ options were enabled [30].

#### 2.2.4. Habitat Dynamics Analysis: Loss, Stable, and Gain

To evaluate the spatial redistribution of suitable habitats rather than merely assessing net changes in total area under climate change, a pixel-by-pixel trajectory analysis was performed for each species using R (v4.4.1; [34]). All raster operations were executed via the ‘terra’ package (v1.7-71), and the resulting ecological metrics were tabulated and visualized utilizing the ‘tidyverse’ suite and ‘ggplot2’.

The input layers consisted of continuous logistic suitability surfaces generated by the MaxEnt algorithm for both the baseline (current) climate and each future climate projection. Because all environmental envelopes were constructed on an identical WGS84 grid size, extent, and spatial resolution, the layers were inherently aligned.

To perform categorical change analysis, each continuous surface was converted into a binary suitable/unsuitable map at a standardized logistic threshold of 0.5 (scores of 0.5 or greater were treated as suitable; [11]). We deliberately applied a fixed threshold of 0.5 to maintain methodological continuity. This ensures direct comparability with established baseline habitat models for sympatric Korean mammals, including the Eurasian otter (*Lutra lutra*), yellow-throated marten (*Martes flavigula*), and long-tailed goral (*Naemorhedus caudatus*) [9,18,23].

**Figure 1:**
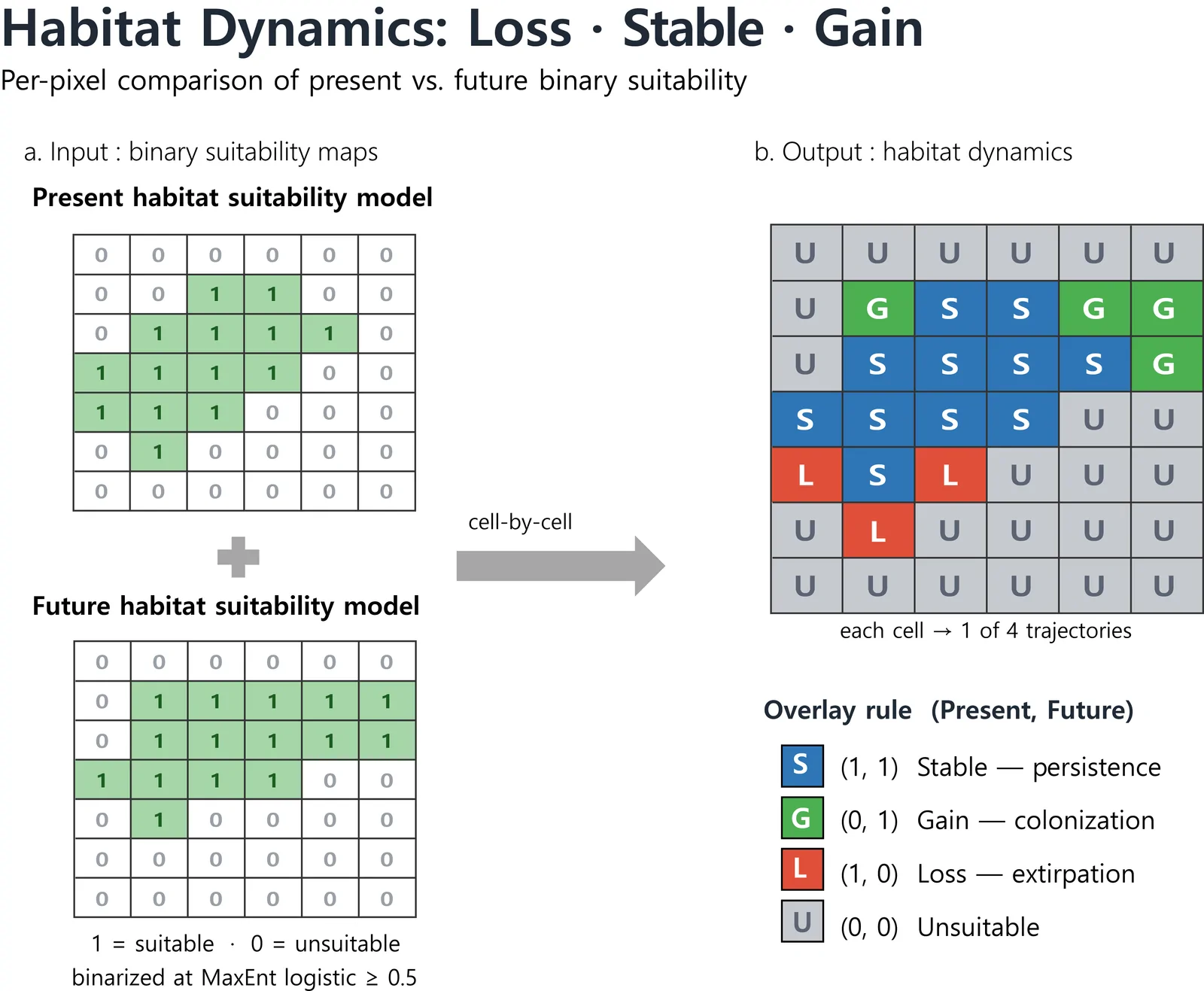
Schematic of the per-pixel habitat-dynamics analysis used to classify changes in suitable habitat between the present and future climates. (**a**) Input layers: the continuous MaxEnt logistic suitability surfaces for the current climate and for each future projection are reclassified into binary maps at a logistic threshold of 0.5, so that every grid cell is scored as suitable (1) or unsuitable (0). (**b**) Output: the present and future binary maps are then compared cell by cell, and each grid cell is assigned to one of four trajectories according to its (present, future) state—Stable/persistence (1, 1), Gain/colonization (0, 1), Loss/extirpation (1, 0), or Unsuitable (0, 0). Net range change is calculated as Gain minus Loss; cells that are unsuitable in both periods (0, 0) are excluded from the habitat metrics. The grids shown are illustrative examples, not empirical results (High-resolution vector schematics are provided in Supplementary Material S1).

For every unique combination of GCM, SSP, and temporal period (5 GCMs × 4 SSPs × 4 periods per species), the binary current distribution map was cross-compared with the corresponding future projection on a per-pixel basis. Each pixel was assigned to one of four dynamic trajectories based on its transitions (Figure 1). ‘Loss’ (suitable at present but unsuitable in the future), ‘Stable’ or ‘Persistence’ (suitable in both periods), ‘Gain’ (unsuitable at present but suitable in the future), and ‘Unsuitable’ (unsuitable across both periods; [35]). Net range change was defined as ‘Gain’ minus ‘Loss’, representing the net percentage variance in total suitable habitat relative to the present.

## 3. Results

In this study, we evaluated and compared the projected changes in habitat suitability for five endangered terrestrial mammal species in the Republic of Korea using CMIP6 climate scenarios across multiple GCMs, SSPs, and future time periods.

### 3.1. Eurasian Otter (Lutra lutra)

Under current climate conditions, the suitable habitat projected for the Eurasian otter (*Lutra lutra*) encompassed 29,867 grid cells, accounting for 21.1% of the total terrestrial extent of the Republic of Korea (141,379 cells). These suitable areas were predominantly distributed along the inland river networks adjacent to the Baekdudaegan Mountain Range and freshwater-inflow zones along the southern coast.

**Figure 2:**
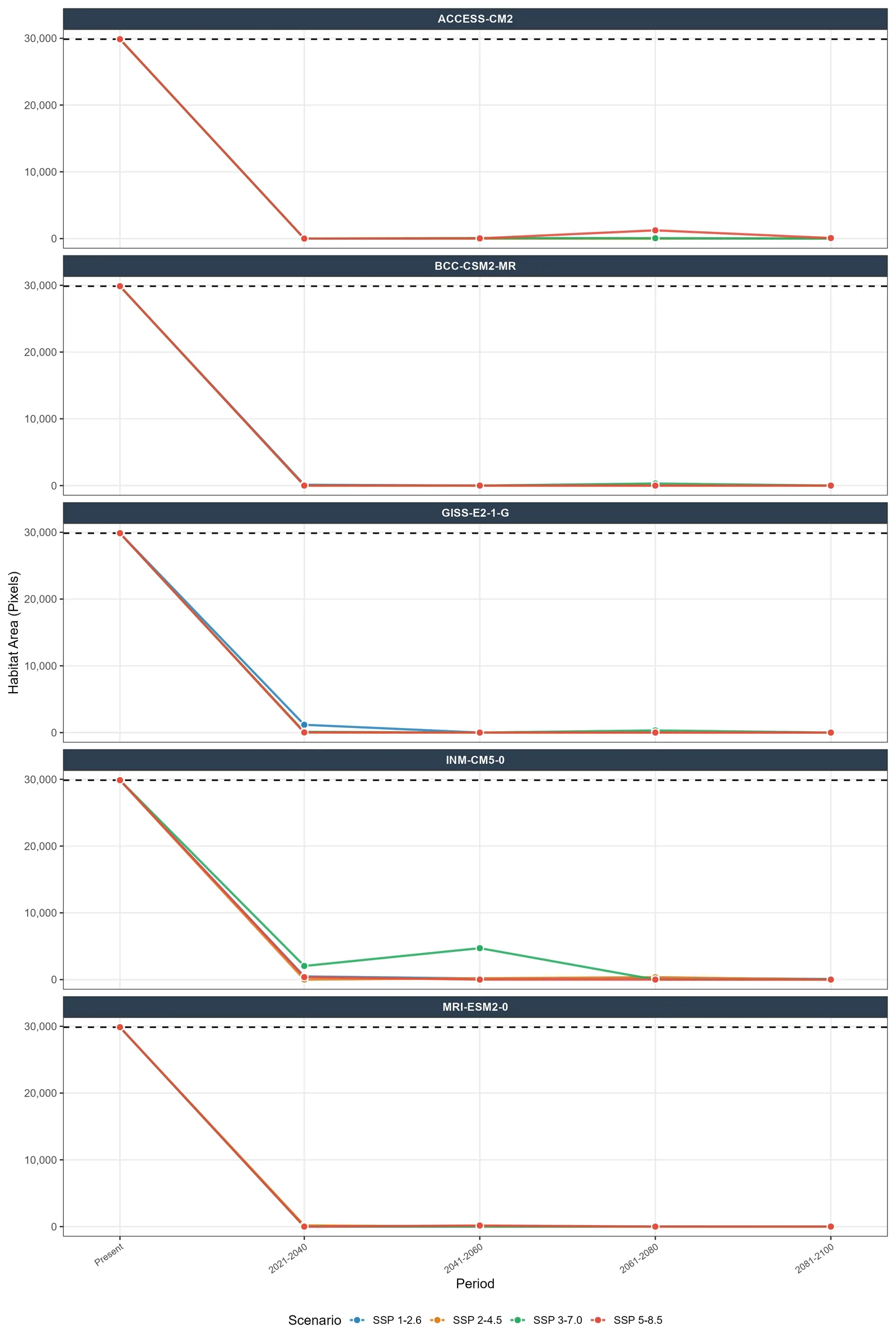
Projected suitable habitat grid-cell counts for *Lutra lutra* under five GCMs (ACCESS-CM2, BCC-CSM2-MR, GISS-E2-1-G, INM-CM5-0, and MRI-ESM2-0) across four SSP scenarios (SSP1–2.6, SSP2–4.5, SSP3–7.0, and SSP5–8.5) and four future time periods (2021–2040, 2041–2060, 2061–2080, and 2081–2100) relative to the current baseline (the horizontal dashed line).

**Figure 3:**
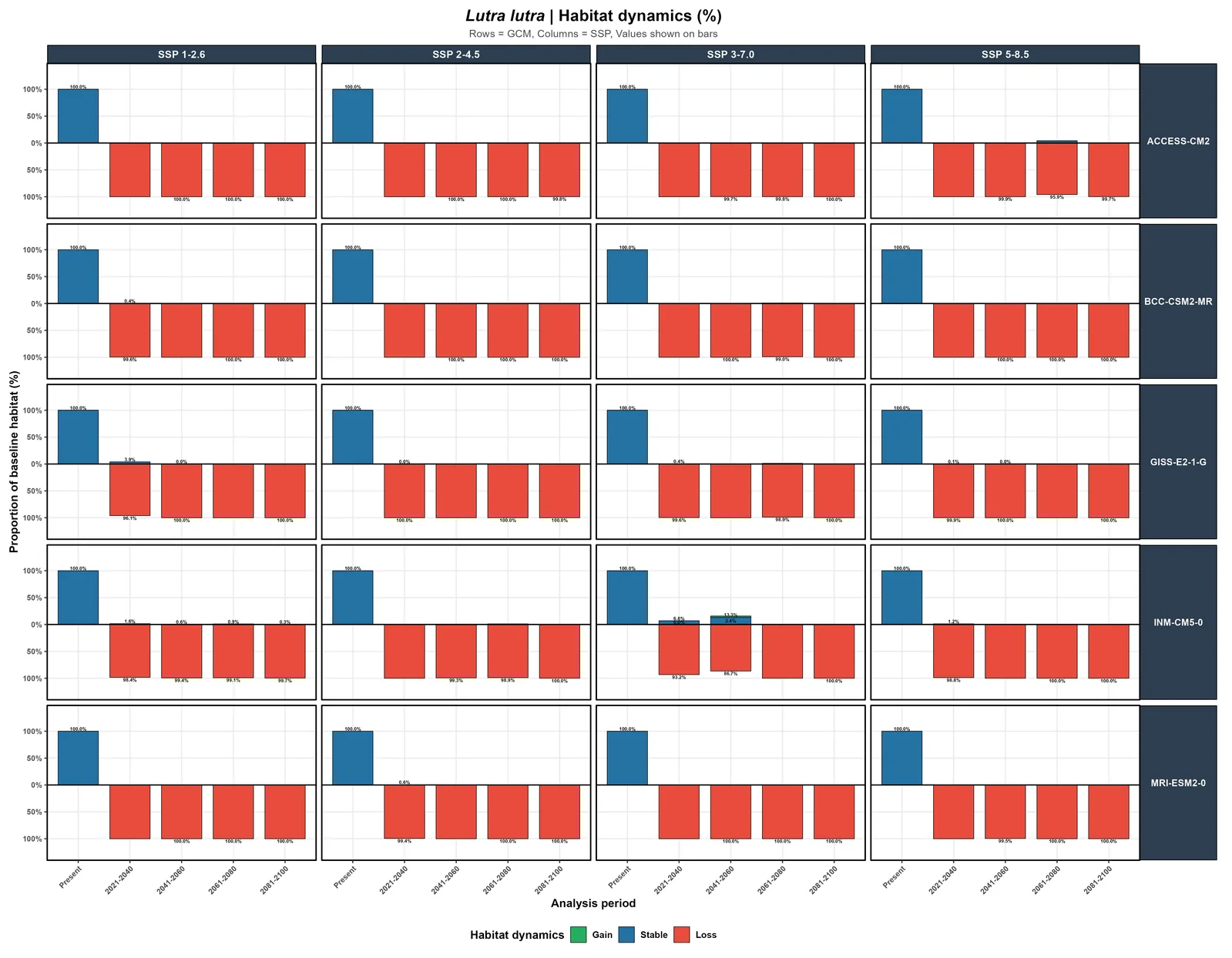
Proportional habitat dynamics (% loss, stable, and gain) for *Lutra lutra* projected by five GCMs across four SSP scenarios (SSP1–2.6, SSP2–4.5, SSP3–7.0, and SSP5–8.5) and four future time periods (2021–2040, 2041–2060, 2061–2080, and 2081–2100) relative to current suitable habitat.

Future bioclimatic projections across all five GCMs indicated a rapid and substantial contraction in the suitable habitat extent of *L. lutra*, initiating from the near-term future (2021–2040) (Figure 2; see Figure S1 for detailed spatial suitability maps across all GCMs). Notably, under the INM-CM5-0 model for the SSP3–7.0 scenario, the suitable area experienced a transient rebound during the 2041–2060 period before declining substantially by 2081–2100 (Figure 2). The magnitude of habitat degradation resulted in a reduction of over 95% relative to the baseline, culminating in complete loss across various scenario-model combinations. When decomposing these dynamics into explicit categories of Loss-Stable-Gain (Figure 3), the proportion of stable habitat patches (Stable) plummeted below 5% from the near future onward, while novel habitat (Gain) was virtually nonexistent (Figure 3).

### 3.2. Yellow-Throated Marten (Martes flavigula)

Under the current climate, the suitable habitat projected for the yellow-throated marten (*Martes flavigula*) encompassed 26,711 grid cells, representing 18.9% of the total terrestrial extent of the Republic of Korea (141,379 cells). Highly suitable habitat patches were predominantly concentrated along the Baekdudaegan Mountain Range and the eastern alpine ecosystems.

**Figure 4:**
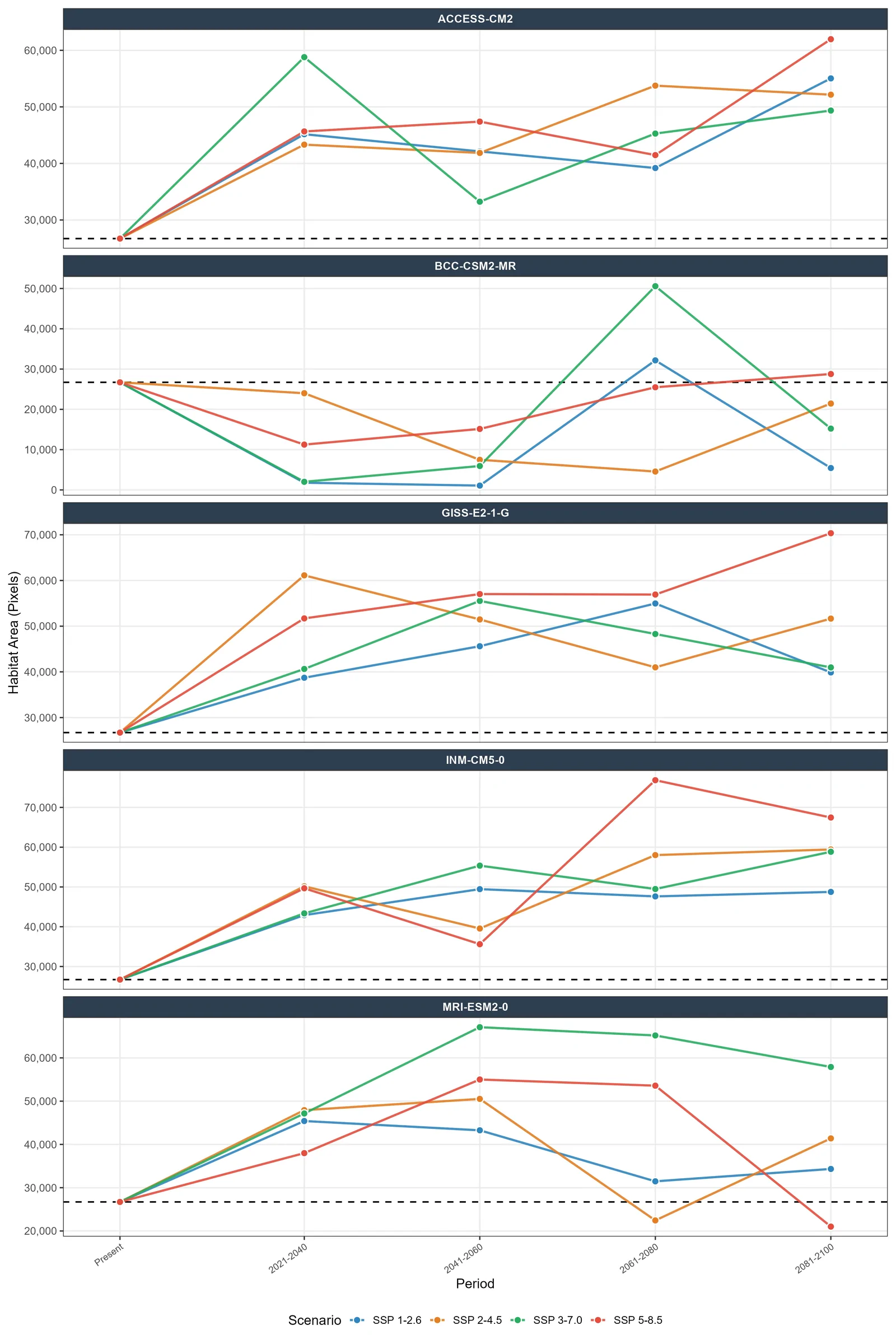
Projected suitable habitat grid-cell counts for *Martes flavigula* under five GCMs (ACCESS-CM2, BCC-CSM2-MR, GISS-E2-1-G, INM-CM5-0, and MRI-ESM2-0) across four SSP scenarios (SSP1–2.6, SSP2–4.5, SSP3–7.0, and SSP5–8.5) and four future time periods (2021–2040, 2041–2060, 2061–2080, and 2081–2100) relative to the current baseline (the horizontal dashed line).

**Figure 5:**
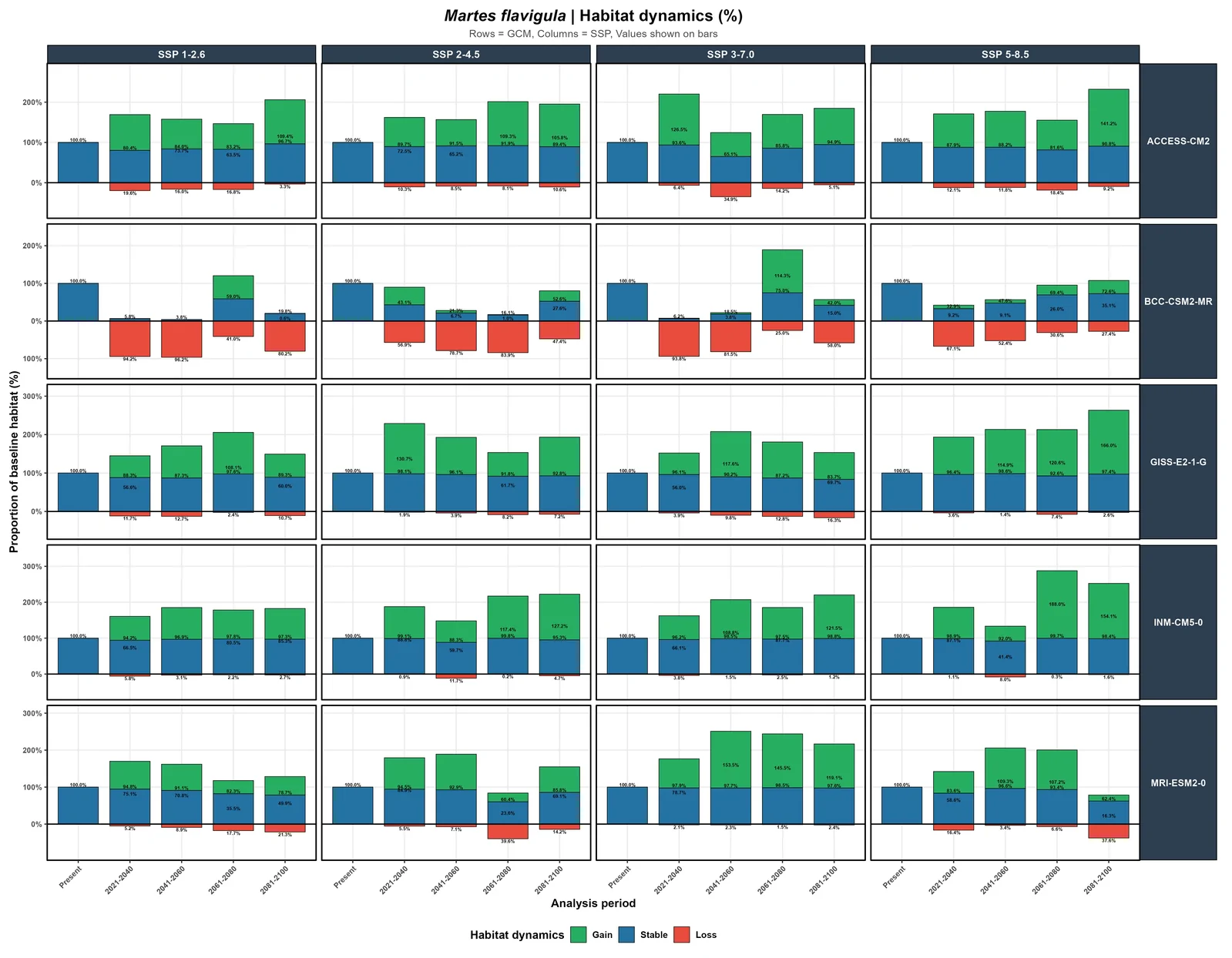
Proportional habitat dynamics (% loss, stable, and gain) for *Martes flavigula* projected by five GCMs across four SSP scenarios (SSP1–2.6, SSP2–4.5, SSP3–7.0, and SSP5–8.5) and four future time periods (2021–2040, 2041–2060, 2061–2080, and 2081–2100) relative to current suitable habitat.

Among the five endangered mammals evaluated in this study, *M. flavigula* was the sole species that demonstrated an expanding trajectory for future suitable habitat across the majority of GCMs (Figure 4; Figure S1). Three specific climate models (ACCESS-CM2, GISS-E2-1-G, and INM-CM5-0) consistently projected substantial area increases ranging from approximately +24% to +188% relative to the current model across all SSPs and future time frames, with MRI-ESM2-0 displaying a similar upward trend. Conversely, the BCC-CSM2-MR model exhibited a starkly conflicting pattern, projecting substantial habitat contractions of −72% to −96% under low-emission scenarios, with the direction of net change reversing between −96% and +89% depending on the scenario-period matrix. Deconstructing these dynamics into explicit spatial components of Loss- Stable -Gain revealed that models predicting overall expansion retained approximately 80–98% of the contemporary habitat as stable patches (Stable) while superimposing substantial novel areas (Gain) equivalent to 57–166% of the current habitat model (Figure 5).

### 3.3. Long-Tailed Goral (Naemorhedus caudatus)

Under current climate conditions, the suitable habitat projected for the long-tailed goral (*Naemorhedus caudatus*) encompassed 4626 grid cells, representing only 3.3% of the total terrestrial extent of the Republic of Korea (141,379 cells). Notably, 97.7% of the presence grids were situated within forested montane regions, and no goral presence was detected south of latitude 36°16′ N.

**Figure 6:**
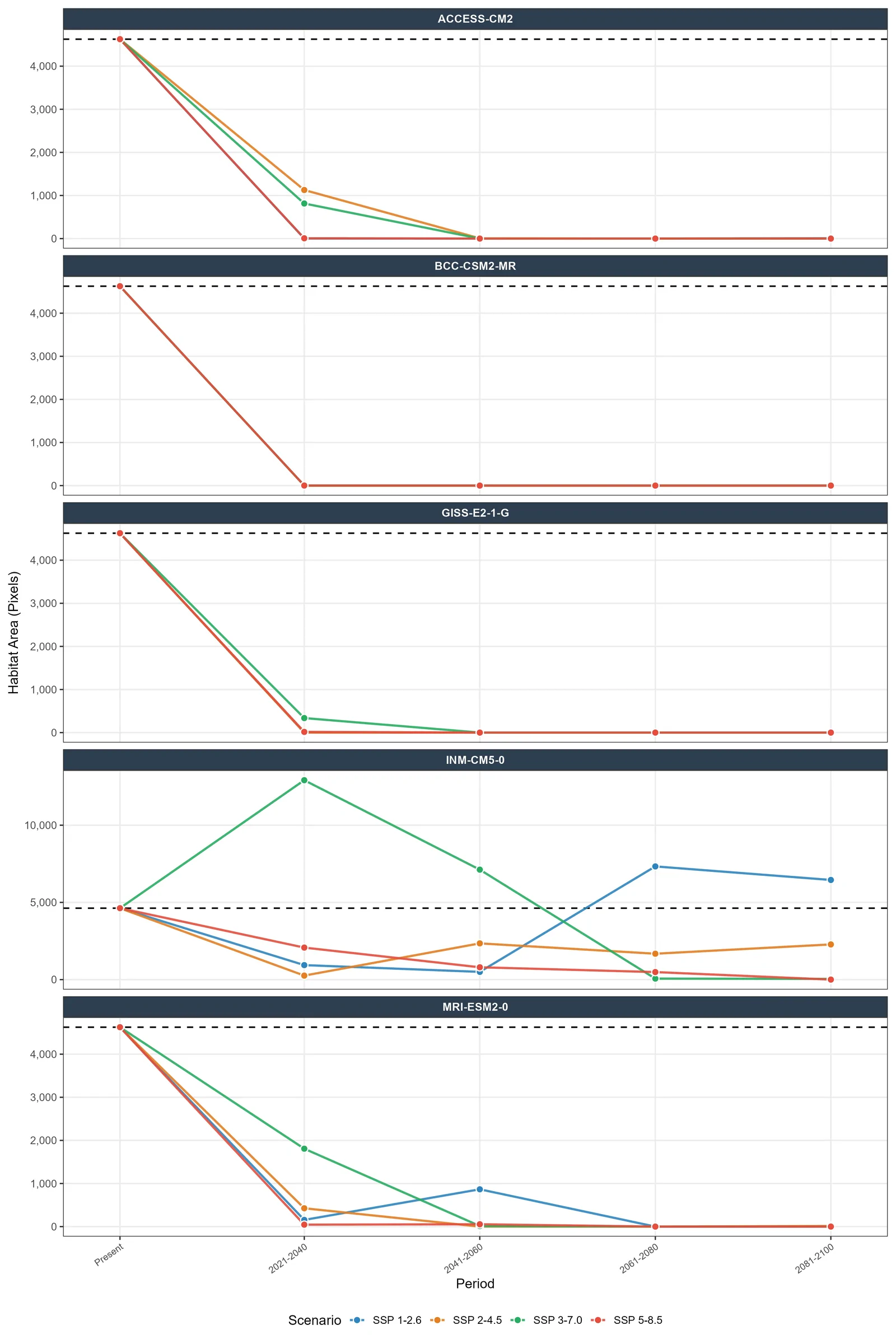
Projected suitable habitat grid-cell counts for *Naemorhedus caudatus* under five GCMs (ACCESS-CM2, BCC-CSM2-MR, GISS-E2-1-G, INM-CM5-0, and MRI-ESM2-0) across four SSP scenarios (SSP1–2.6, SSP2–4.5, SSP3–7.0, and SSP5–8.5) and four future time periods (2021–2040, 2041–2060, 2061–2080, and 2081–2100) relative to the current baseline (the horizontal dashed line).

**Figure 7:**
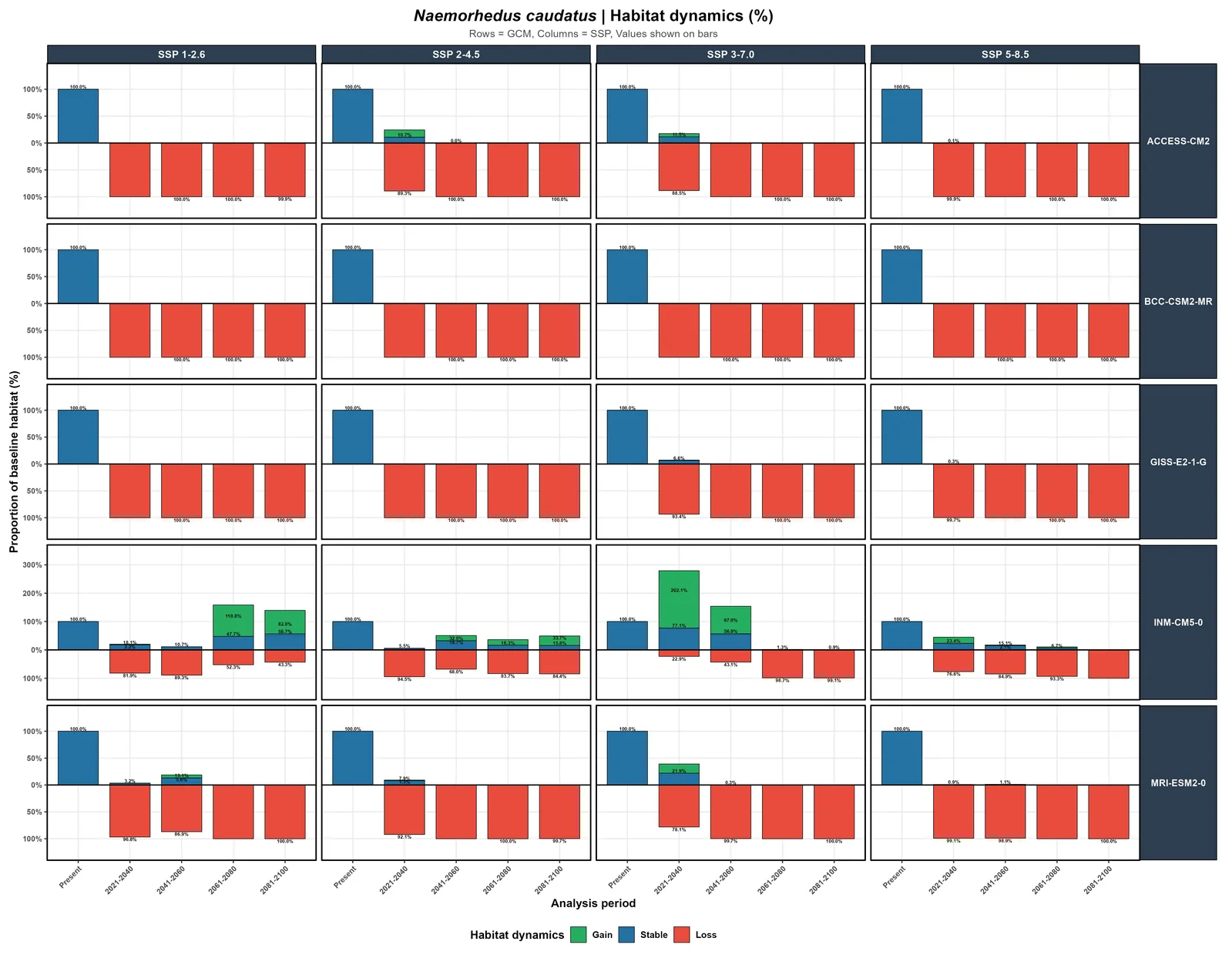
Proportional habitat dynamics (% loss, stable, and gain) for *Naemorhedus caudatus* projected by five GCMs across four SSP scenarios (SSP1–2.6, SSP2–4.5, SSP3–7.0, and SSP5–8.5) and four future time periods (2021–2040, 2041–2060, 2061–2080, and 2081–2100) relative to current suitable habitat.

Future projections of the suitable habitat area for *Naemorhedus caudatus* revealed a rapid, severe, and consistent contraction across four GCMs (ACCESS-CM2, BCC-CSM2-MR, GISS-E2-1-G, and MRI-ESM2-0), showing declines of −60% to −100% starting from the near-term future, with models projecting substantial contraction of suitable thermal envelopes under long-term high-emission scenarios (Figure 6; Figure S1). The sole outlier showing a divergent trajectory was the INM-CM5-0 model, which projected a transient, short-term expansion of approximately +179% relative to the current model during the near-term period under the SSP3–7.0 scenario, before plunging sharply by −99% to −100% in the mid- and long-term horizons. Deconstructing these patterns into explicit Loss-Stable-Gain dynamics indicated that for the four consensus GCMs, stable habitat patches (Stable) contracted to less than 3% from the near future onward, with spatial loss dominating the range dynamics (97–100%, Figure 7).

### 3.4. Leopard Cat (Prionailurus bengalensis)

Under current climate conditions, the suitable habitat projected for the leopard cat (*Prionailurus bengalensis*) encompassed 51,136 grid cells, accounting for 36.2% of the total terrestrial extent of the Republic of Korea (141,379 cells). These suitable patches were widely distributed across the western coastal plains, spanning the entire middle and southern regions, as well as the southern coast.

**Figure 8:**
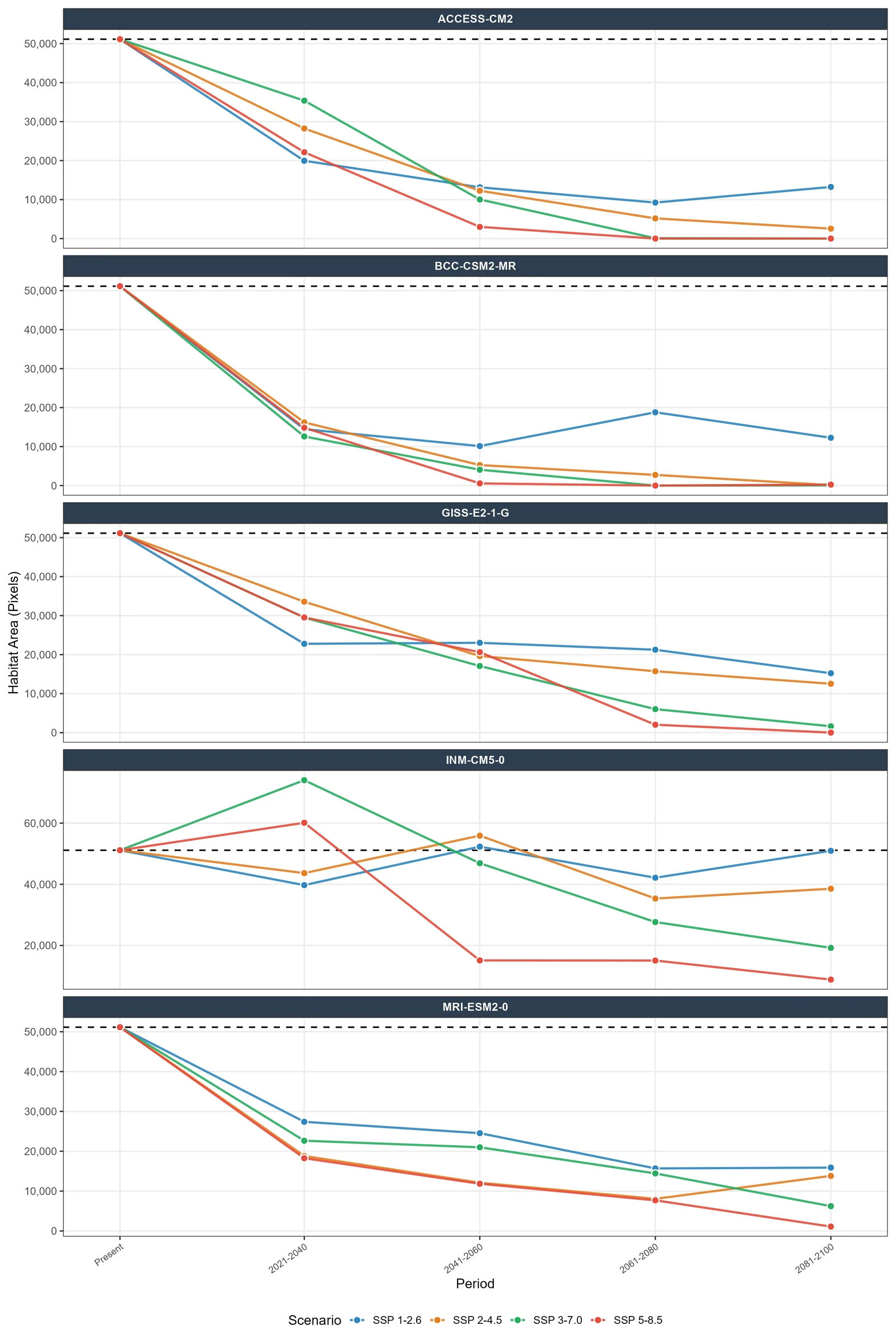
Projected suitable habitat grid-cell counts for *Prionailurus bengalensis* under five GCMs (ACCESS-CM2, BCC-CSM2-MR, GISS-E2-1-G, INM-CM5-0, and MRI-ESM2-0) across four SSP scenarios (SSP1–2.6, SSP2–4.5, SSP3–7.0, and SSP5–8.5) and four future time periods (2021–2040, 2041–2060, 2061–2080, and 2081–2100) relative to the current baseline (the horizontal dashed line).

**Figure 9:**
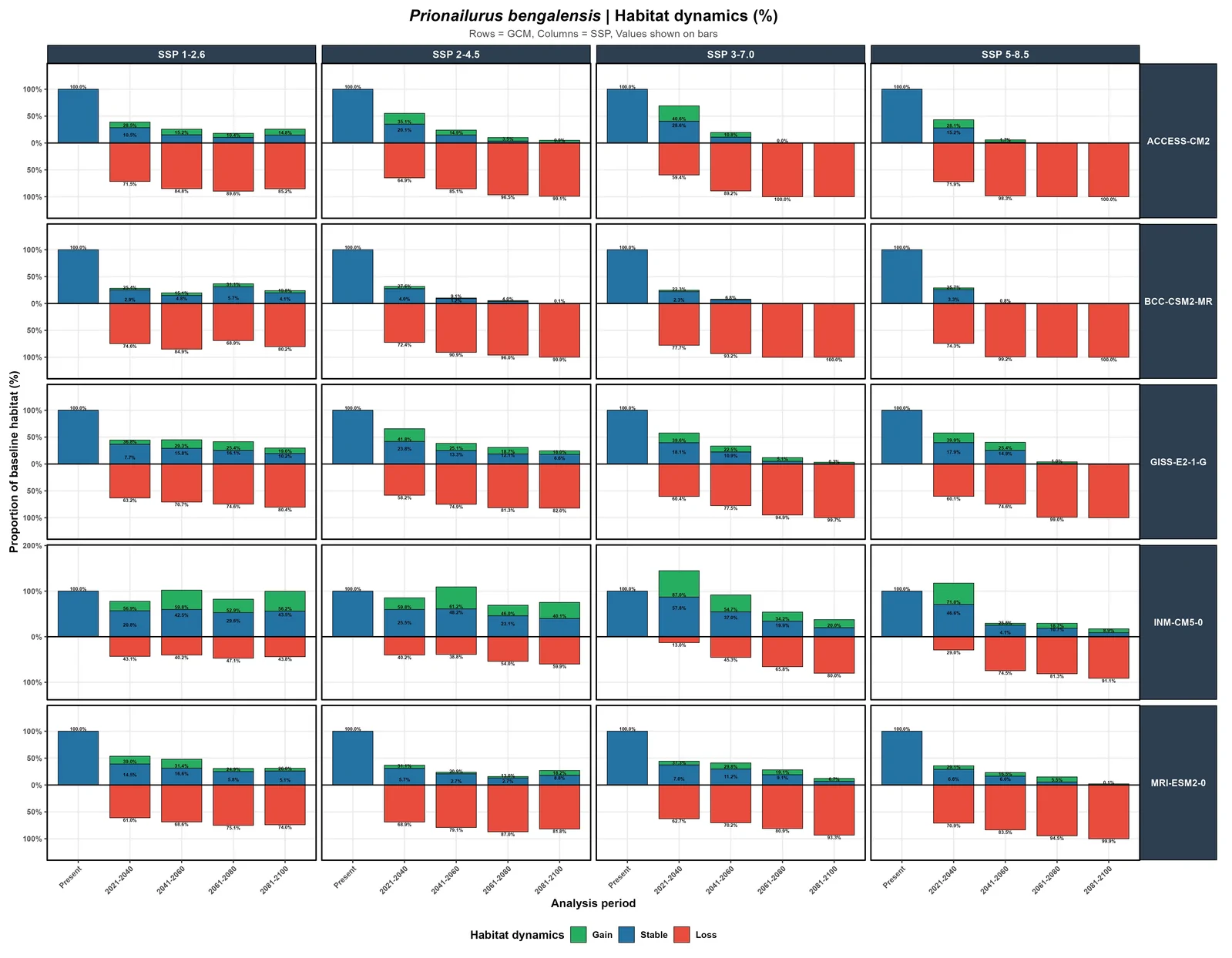
Proportional habitat dynamics (% loss, stable, and gain) for *Prionailurus bengalensis* projected by five GCMs across four SSP scenarios (SSP1–2.6, SSP2–4.5, SSP3–7.0, and SSP5–8.5) and four future time periods (2021–2040, 2041–2060, 2061–2080, and 2081–2100) relative to current suitable habitat.

Future bioclimatic simulations revealed a gradual and progressive contraction of the suitable habitat area for *P. bengalensis* across four GCMs (ACCESS-CM2, BCC-CSM2-MR, GISS-E2-1-G, and MRI-ESM2-0), with projected declines ranging from −31% to −100% relative to the current model (Figure 8; Figure S1). The sole outlier demonstrating a divergent trajectory was the INM-CM5-0 model, which projected a transient near-term increase of +2% to +45% under certain scenarios before transitioning into pronounced mid- and long-term declines of −60% to −83%. When decomposing these range shifts into explicit Loss-Stable-Gain, the four consensus GCMs retained stable habitat patches (Stable) at approximately 25–39% in the near-term future, but ultimately converged toward 100% spatial loss over extended time horizons (Figure 9).

### 3.5. Siberian Flying Squirrel (Pteromys volans)

Under current climate conditions, the suitable habitat projected for the Siberian flying squirrel (*Pteromys volans*) encompassed 31,137 grid cells, representing 22.0% of the total terrestrial extent of the Republic of Korea (141,379 cells). Highly suitable habitat patches were predominantly clustered along the core mountain ridges of the Baekdudaegan Mountain Range and the montane ecosystems of Gangwon Province.

**Figure 10:**
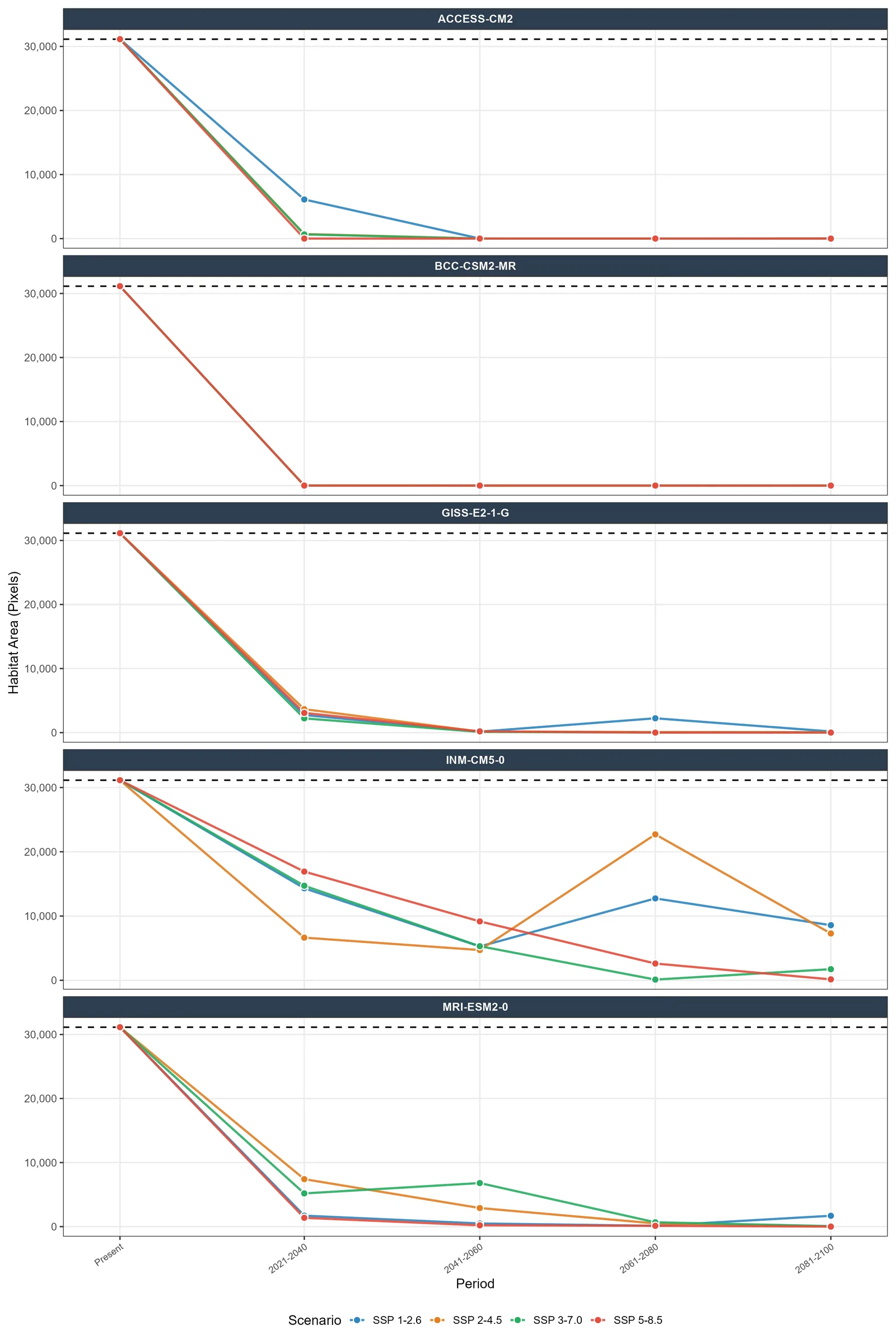
Projected suitable habitat grid-cell counts for *Pteromys volans* under five GCMs (ACCESS-CM2, BCC-CSM2-MR, GISS-E2-1-G, INM-CM5-0, and MRI-ESM2-0) across four SSP scenarios (SSP1–2.6, SSP2–4.5, SSP3–7.0, and SSP5–8.5) and four future time periods (2021–2040, 2041–2060, 2061–2080, and 2081–2100) relative to the current baseline (the horizontal dashed line).

**Figure 11:**
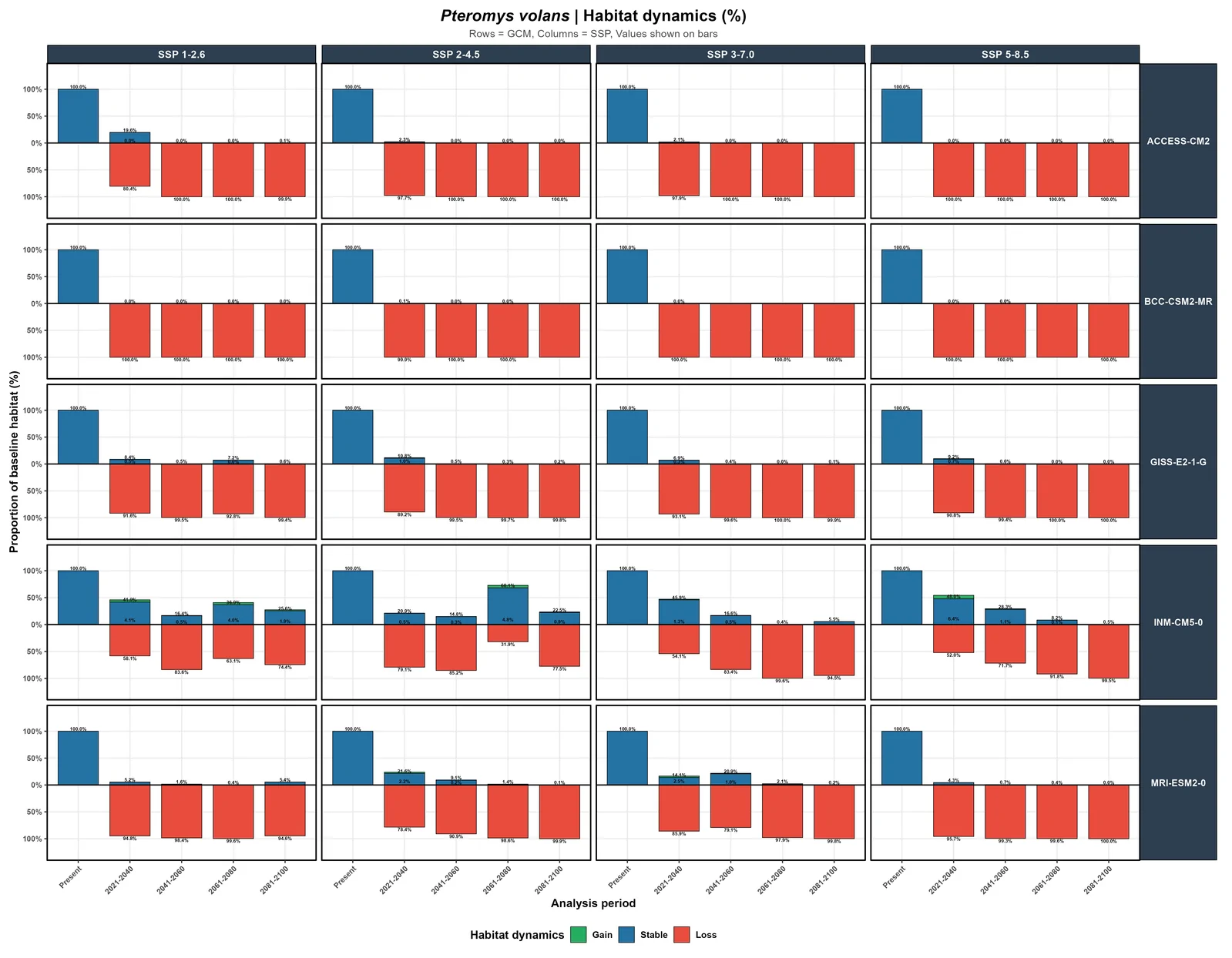
Proportional habitat dynamics (% loss, stable, and gain) for *Pteromys volans* projected by five GCMs across four SSP scenarios (SSP1–2.6, SSP2–4.5, SSP3–7.0, and SSP5–8.5) and four future time periods (2021–2040, 2041–2060, 2061–2080, and 2081–2100) relative to current suitable habitat.

Future bioclimatic simulations revealed an immediate, severe, and consistent contraction of the suitable habitat area for P. volans across four GCMs (ACCESS-CM2, BCC-CSM2-MR, GISS-E2-1-G, and MRI-ESM2-0), showing projected declines of −76% to −100% starting from the near-term future (2021–2040) (Figure 10; Figure S1). Even under the low emission pathway (SSP1–2.6), the suitability area decreased by over 80%. The INM-CM5-0 model acted as the sole outlier, projecting a relatively moderate downward trajectory of −27% to −85% during the near- to mid-term periods, though it ultimately converged toward extensive habitat collapse (−92% to −100%) under long-term high-emission configurations (Figure 10). Deconstructing these shifts into explicit spatial components indicated that the four consensus models simulated a drop in stable patches (Stable) to 0–20% from the near future onward (Figure 11).

## 4. Discussion

By deconstructing the spatial dynamics of suitable habitats into explicit categories of Loss, Stable, and Gain for each endangered species, we discuss the profound implications of these range shifts for proactive wildlife conservation and climate-resilient spatial management planning. Because our predictive framework is modeled primarily on macroclimatic constraints and topographic covariates, these projections should be interpreted as a spatial quantification of potential vulnerability ranges rather than direct population dynamics, thereby serving as a rigorous scientific foundation for long-term conservation prioritization [36].

### 4.1. Ecological Traits and Climate Vulnerability of Target Taxa

The contrasting trajectories projected across species reflect differences in their underlying ecological traits and macroecological range positions [3].

#### 4.1.1. Eurasian Otter (*Lutra lutra*)

As a semiaquatic predator, *L. lutra* relies mainly on fishes, while the dietary proportions of secondary prey, such as amphibians, insects, and crustaceans, vary depending on the size and geomorphic type of the inhabited river systems [37,38]. These diet compositions and home-range dimensions exhibit individual variation by food availability and hydro-geomorphic river typologies [39,40,41,42,43,44]. Landscape scale assessments in the Republic of Korea identified land cover types, water quality parameters, and anthropogenic disturbances as key determinants of habitat selection [9]. Such intimate ecological dependence on highly specific riparian environments and associated trophic resources makes *L. lutra* exceptionally vulnerable to climate change. Climate-driven alterations in stream temperature and hydrological dynamics directly compromise fish and crustacean community structures [45,46], which subsequently cascades through the aquatic food web to drive widespread habitat degradation for otters [47].

#### 4.1.2. Yellow-Throated Marten (*Martes flavigula*)

Fulfilling pivotal ecological roles as both an apex predator and an effective seed disperser within forest ecosystems, *M. flavigula* is an agile, wide-ranging omnivorous carnivore that utilizes a diverse array of trophic resources including small mammals, birds, reptiles, and wild fruits [40]. Due to their expansive home ranges and high activity patterns, their habitat utilization remains intimately bound to mature forest structures [48]. Landscape-scale analyses on South Korean populations have previously demonstrated that forest cover types, altitudinal gradients, and traffic volumes act as compounding determinants governing macro-geographic patch selection, confirming its status as a forest-specialist indicator [18]. Biogeographically, the core distribution of this species stretches across tropical and subtropical Asia, placing the Korean Peninsula at its northernmost range margin [40,48]. Populations positioned at such high-latitude leading edges frequently exhibit range expansions in response to global warming as higher altitudes become bioclimatically permissive [3]. Consequently, the multi-model consensus toward increased climate suitability for *M. flavigula* observed here strongly conforms to established macroecological core-periphery range-shift dynamics.

#### 4.1.3. Long-Tailed Goral (*Naemorhedus caudatus*)

The long-tailed goral strictly inhabits steep cliffs, rugged rocky terrains, and mixed coniferous-deciduous forests, feeding primarily on herbaceous plants and shrubs [40]. Due to their low dispersal capacity and high topographic dependency, their spatial utilization patterns have been reported to react sensitively to extreme weather anomalies, such as heavy snowfall events [49]. The vulnerability of *N. caudatus* stems from the compounding effects of its physiological preference for cold boreal climates and substantial topographic restrictions on movement. Because alpine zones exceeding 1000 m in altitude are extremely limited in the Republic of Korea, climate-driven upslope shifts of cold thermal envelopes substantially reduce total available land area [50,51], exposing the goral to mountaintop extirpation risks characteristically documented in alpine mammals [52]. Furthermore, alterations in snowfall regimes and the shifting distribution of forage vegetation are likely to aggravate habitat quality within currently occupied altitudinal ranges [49]. Such extensive range contractions and structural isolation could significantly accelerate genetic erosion via inbreeding depression and random genetic drift [53,54,55].

#### 4.1.4. Leopard Cat (*Prionailurus bengalensis*)

The leopard cat operates as a small, opportunistic carnivore that exploits a highly diverse dietary niche consisting of small mammals, birds, and reptiles, utilizing a broad spectrum of environments ranging from riparian-wetland systems to agricultural-forest ecotones [40,56]. It displays significant behavioral plasticity in home range dimensions and patch preference across seasons and sexes [57]. Although current suitable habitats for the leopard cat are heavily concentrated within low-altitude landscapes [58,59], progressive regional warming that renders these lowlands bioclimatically unsuitable leaves localized populations with limited high-altitude refugia to colonize, exposing the species to risks of lowland biotic attrition [60].

#### 4.1.5. Siberian Flying Squirrel (*Pteromys volans*)

The Siberian flying squirrel operates as an obligate arboreal specialist strictly dependent on mature, old-growth deciduous forests with high canopy connectivity, relying primarily on tree cavities in old trees for nesting, breeding, and predator avoidance [40,61]. In the Republic of Korea, empirical field surveys indicate that the highest occurrence densities and feeding signs are concentrated within old-growth forests adjacent to riparian corridors [62,63,64]. Macroecological studies demonstrate that background climate acts as a principal driver alongside microhabitat availability and predation pressure governing species occurrence [65]. As suitable bioclimatic envelopes for old-growth forest formations shift upslope toward higher altitudes, geometric constraints of mountainous topography dictate that available land area substantially narrows with increasing elevation, thereby severely reducing total available habitat extent [51]. Furthermore, when mature forest cover drops below critical thresholds, occupancy probability collapses non-linearly [66].

### 4.2. Model Uncertainties and GCM Variations

In addition to these ecological constraints, prominent inter-model variations introduced across alternative GCM architectures underscore the critical danger of relying on single-GCM frameworks for space-based decision making [13].

For instance, the short-term habitat expansion or transient mitigation simulated by the INM-CM5-0 model across several species (*L. lutra*, *N. caudatus*, *P. bengalensis*, and *P. volans*) is principally driven by its lower Equilibrium Climate Sensitivity (ECS), which dictates a slower rate of simulated contemporary warming [27]. Given that this same model projects a substantial contraction in subsequent mid- and long-term horizons, these transient signals represent temporary model artifacts rather than functional habitat gains [15]. Similarly, the prominent inter-model uncertainty introduced by the BCC-CSM2-MR projection for *M. flavigula* highlights the necessity of multi-model ensemble forecasting to account for predictive uncertainties [13,15].

### 4.3. Conservation Priorities and Spatial Management

To minimize risk under future climate uncertainty, proactive spatial policies must move beyond passive reliance on volatile bioclimatic projections [15,18]. Based on our ensemble modeling and empirical literature, we highlight three core management imperatives.

#### 4.3.1. Legal Reinforcement of Refugia

Proactive spatial policies should prioritize the legal reinforcement and protection of verified contemporary core habitats across the Baekdudaegan Mountain Range and eastern mountain massifs (such as the Seorak–Odae mountain complex) as primary climate refugia [15,18,23,67,68].

#### 4.3.2. Structural Corridors

Implementing structural mitigation measures, including wildlife crossing corridors and the removal of infrastructure barriers, represents a vital management imperative to safeguard landscape connectivity, maintain gene flow, and sustain long-term ecosystem integrity [15,57,69].

#### 4.3.3. Microhabitat Restoration

For species dependent on specific structural features, such as *P. volans*, strict legal protection of mature riparian deciduous forests and active implementation of artificial nest boxes and canopy corridors are essential to bridge fragmenting patches and maintain functional connectivity across the species’ range [62].

## 5. Conclusions

This study underscores the critical importance of multi-model ensemble frameworks in forecasting the contrasting, species-specific range dynamics of endangered terrestrial mammals under global climate change. Our macroclimatic simulations demonstrate that while the yellow-throated marten may experience bioclimatic range expansion on the Korean Peninsula due to high latitude range-edge dynamics, the remaining four protected taxa—the Eurasian otter, leopard cat, long-tailed goral, and Siberian flying squirrel—face substantial, progressive habitat contractions leading to near-complete regional loss under high-emission pathways. Furthermore, prominent inter-model anomalies and variations across alternative global climate model architectures highlight the inherent uncertainty embedded within future projections, reinforcing that volatile transient signals should not be interpreted with undue optimism.

Crucially, our findings highlight a fundamental distinction between projected bioclimatic suitability envelopes and actual habitat occupancy. In real-world landscapes, functional occupancy will be heavily bottlenecked by non-climatic anthropogenic stressors, including extensive regional road networks, border fence systems, and an escalation in climate-driven extensive wildfires.

Importantly, as our MaxEnt modeling framework relies primarily on macroclimatic covariates and elevation, the projected bioclimatic envelopes represent potential thermal suitability rather than real-world functional occupancy. In real-world landscapes, species persistence will be further bottlenecked by non-climatic anthropogenic stressors that were not directly parameterized in this study, including dense regional road networks, African Swine Fever (ASF) barrier fence systems, and climate-driven severe wildfires. Integrating these non-climatic landscape barriers with bioclimatic suitability models represents a critical priority for future research to refine realized habitat predictions.

Consequently, rather than relying solely on shifting bioclimatic envelopes, proactive spatial conservation policies should prioritize the legal reinforcement and expansion of protected area networks across verified contemporary core habitats along the Baekdudaegan Mountain Range and the Seorak–Odae mountain complex as primary long-term climate refugia. Alongside spatial protection, targeted infrastructure mitigation is essential, such as installing functional overpass wildlife crossings along high-density traffic axes to reduce vehicle-induced mortality and restore gene flow for wide-ranging species. Microhabitat management must also be actively integrated into regional planning. Preserving mature riparian deciduous forests and installing artificial nest box networks with canopy corridors will critically bridge fragmenting patches for arboreal specialists. Combining multi-model climate modeling with these spatially explicit, taxon-specific management interventions provides an essential framework to safeguard landscape connectivity and sustain long-term ecosystem integrity under accelerating global change.

## Figures and Tables

**Table 1:** Environmental variables used for future projections of habitat suitability for wildlife species in the Republic of Korea based on MaxEnt modeling (adapted from [18,23]).

No	Variable	Description	Source
1	BIO1	Annual mean temperature	https://www.worldclim.org/
2	BIO2	Mean diurnal range [mean of monthly (max temp–min temp)]	https://www.worldclim.org/
3	BIO3	Isothermality (BIO2/BIO7) (×100)	https://www.worldclim.org/
4	BIO4	Temperature seasonality (standard deviation × 100)	https://www.worldclim.org/
5	BIO5	Max temperature of warmest month	https://www.worldclim.org/
6	BIO6	Min temperature of coldest month	https://www.worldclim.org/
7	BIO7	Temperature annual range (BIO5–BIO6)	https://www.worldclim.org/
8	BIO8	Mean temperature of wettest quarter	https://www.worldclim.org/
9	BIO9	Mean temperature of driest quarter	https://www.worldclim.org/
10	BIO10	Mean temperature of warmest quarter	https://www.worldclim.org/
11	BIO11	Mean temperature of coldest quarter	https://www.worldclim.org/
12	BIO12	Annual precipitation	https://www.worldclim.org/
13	BIO13	Precipitation of wettest month	https://www.worldclim.org/
14	BIO14	Precipitation of driest month	https://www.worldclim.org/
15	BIO15	Precipitation seasonality (coefficient of variation)	https://www.worldclim.org/
16	BIO16	Precipitation of wettest quarter	https://www.worldclim.org/
17	BIO17	Precipitation of driest quarter	https://www.worldclim.org/
18	BIO18	Precipitation of warmest quarter	https://www.worldclim.org/
19	BIO19	Precipitation of coldest quarter	https://www.worldclim.org/
20	DEM	Digital elevation model	https://egis.me.go.kr/

BIO1–BIO19 datasets were compiled from WorldClim v2.1 at a 30 arc-second resolution. Digital Elevation Model (DEM) was acquired from the Environmental Geographic Information Service (EGIS). The selection and categorization of environmental covariates follow the standard spatial modeling frameworks established by [19,23].

## Data Availability

The bioclimatic variables and digital elevation model utilized in this study are publicly available from WorldClim (https://www.worldclim.org) and the Environmental Geographic Information Service (https://egis.me.go.kr). The wildlife occurrence datasets were compiled from the National Institute of Biological Resources (NIBR) and the National Institute of Ecology (NIE). Due to institutional regulations and ethical restrictions regarding the precise spatial coordinates of legally protected endangered species, the raw occurrence datasets are not publicly archived but are available from the corresponding author upon reasonable request.

## Supplementary Materials

The following supporting information can be downloaded at: https://www.mdpi.com/article/10.3390/ani16162489/s1, Figure S1: Comprehensive Habitat Suitability Maps for Five Endangered Terrestrial Mammals in Republic of Korea under CMIP6 Climate Change Scenarios, Table S1: Summary of occurrence record counts for five endangered terrestrial mammal species in Republic of Korea before and after spatial autocorrelation filtering; Table S2: Predictive performance metrics (AUC, Omission Rate, and TSS) for MaxEnt models of five endangered terrestrial mammal species in Republic of Korea.
