# Supplementary material for: Projected Future Habitat Suitability of Five Endangered Terrestrial Mammals in South Korea Under CMIP6 Climate Scenarios
Source: Animals (Basel). 2026 Aug 10;16(16):2489. doi: 10.3390/ani16162489 (PMC13508954; doi:10.3390/ani16162489)

Lutra lutra

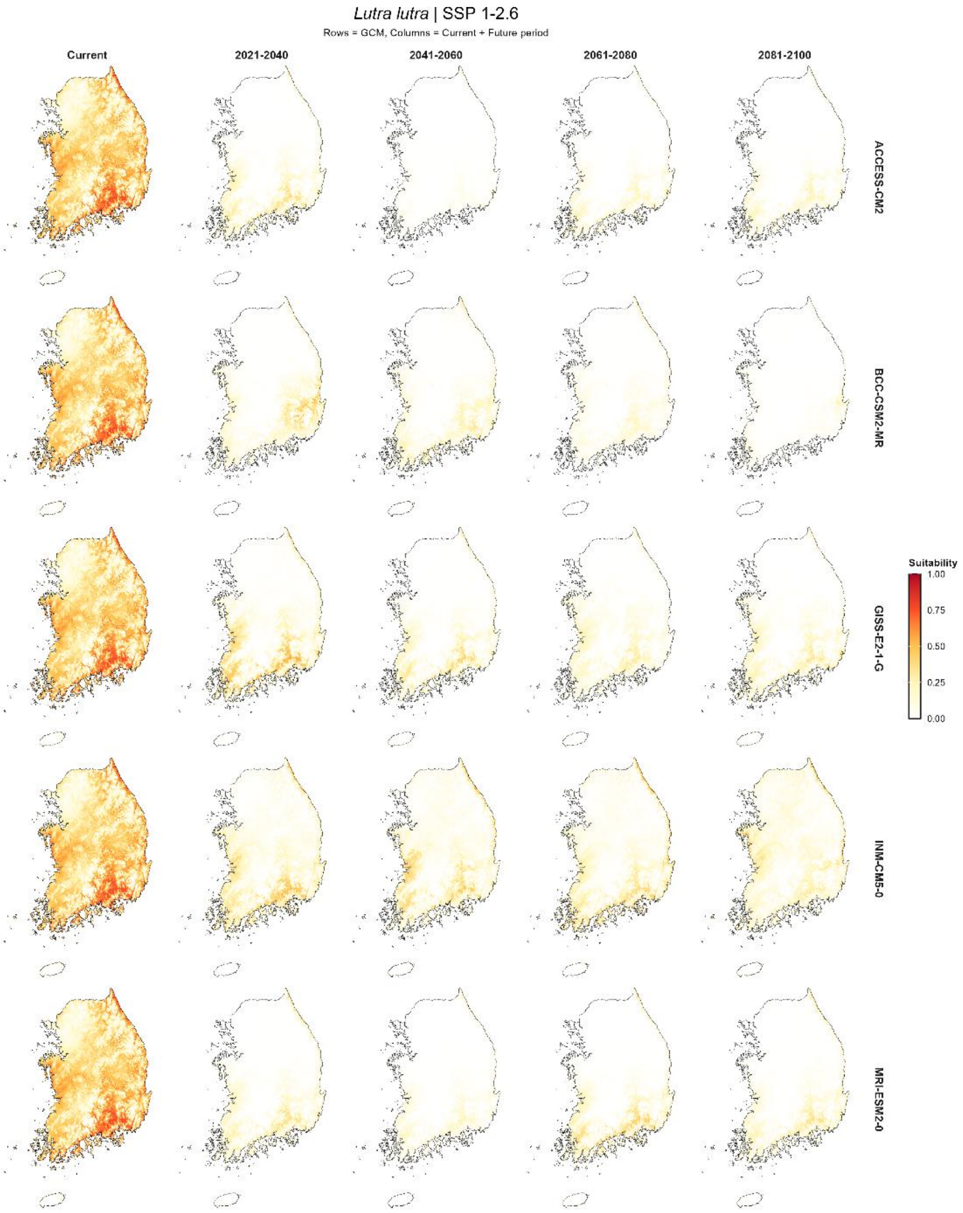
Lutra lutra | SSP 1-2.6
Rows = GCM, Columns = Current + Future period
Current
2021-2040
2041-2060
2061-2080
2081-2100
ACCESS-CM2
BCC-CSM2-MR
GISS-E2-1-G
INM-CM5-0
MRI-ESM2-0
Suitability
1.00
0.75
0.50
0.25
0.00

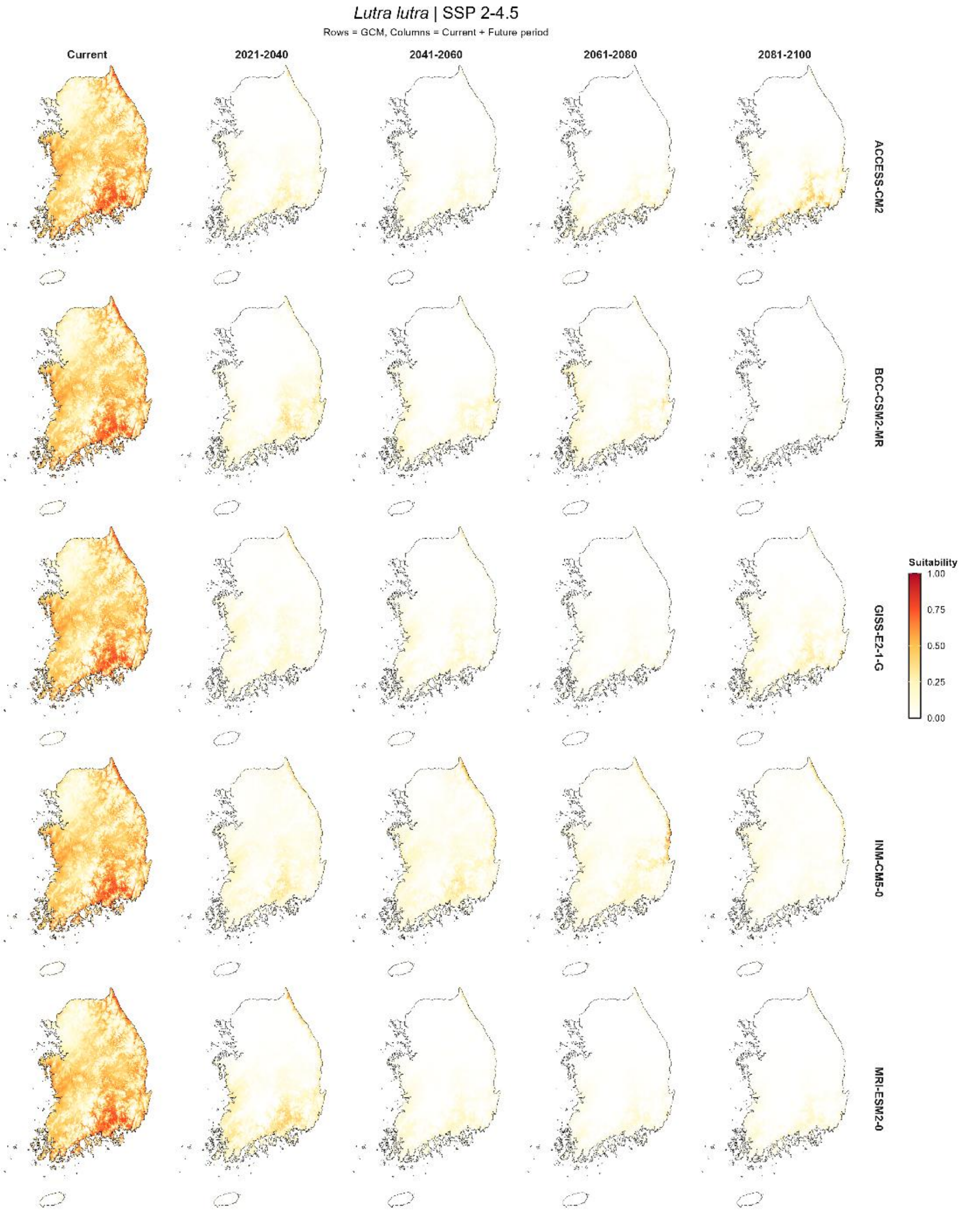
Lutra lutra | SSP 2-4.5
Rows = GCM, Columns = Current + Future period
Current
2021-2040
2041-2060
2061-2080
2081-2100
ACCESS-CM2
BCC-CSM2-MR
GISS-E2-1-G
INM-CM5-0
MRI-ESM2-0
Suitability
1.00
0.75
0.50
0.25
0.00

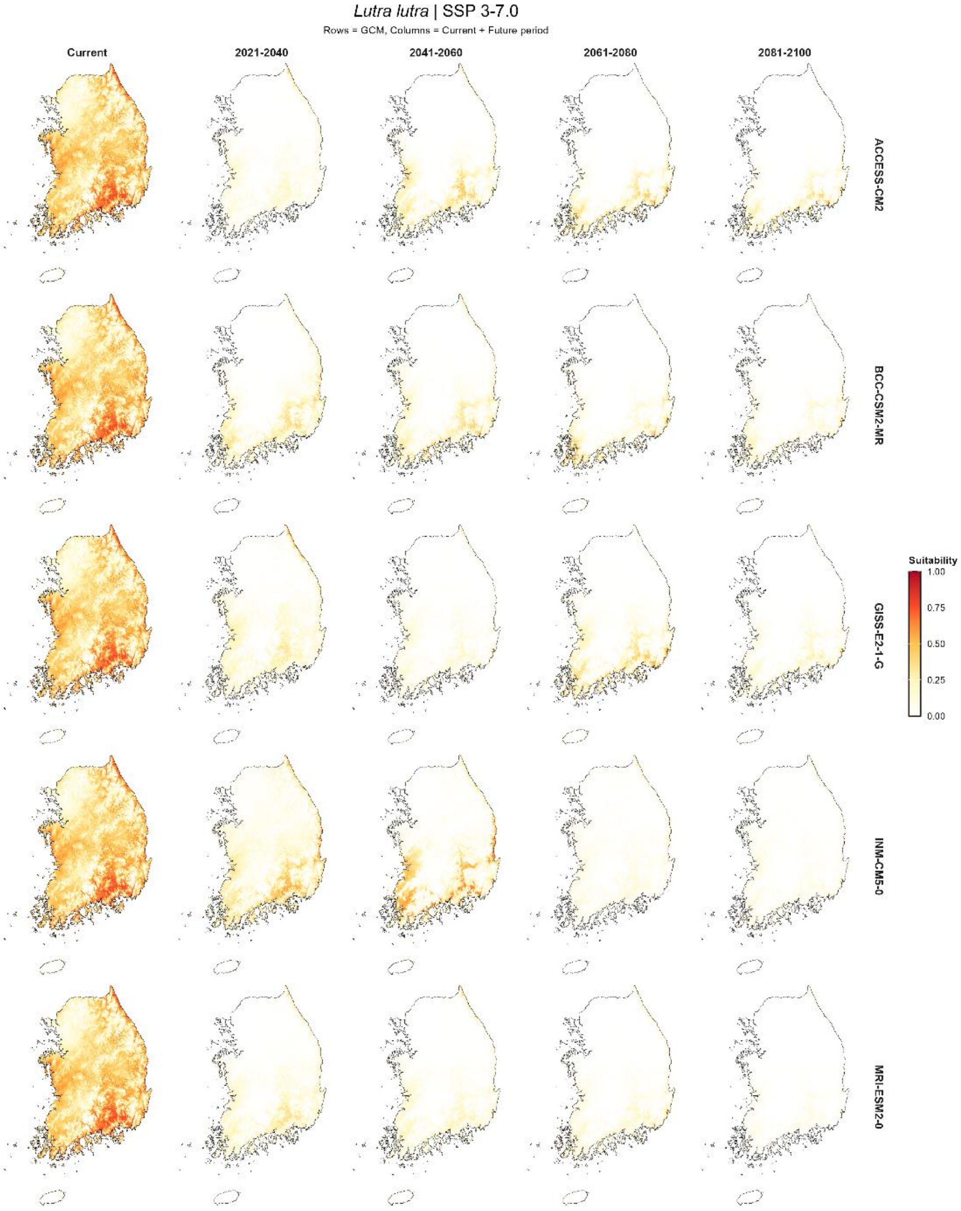

Lutra lutra | SSP 3-7.0
Rows = GCM, Columns = Current + Future period
Current
2021-2040
2041-2060
2061-2080
2081-2100
ACCESS-CM2
BCC-CSM2-MR
GISS-E2-1-G
INM-CM5-0
MRI-ESM2-0
Suitability
1.00
0.75
0.50
0.25
0.00

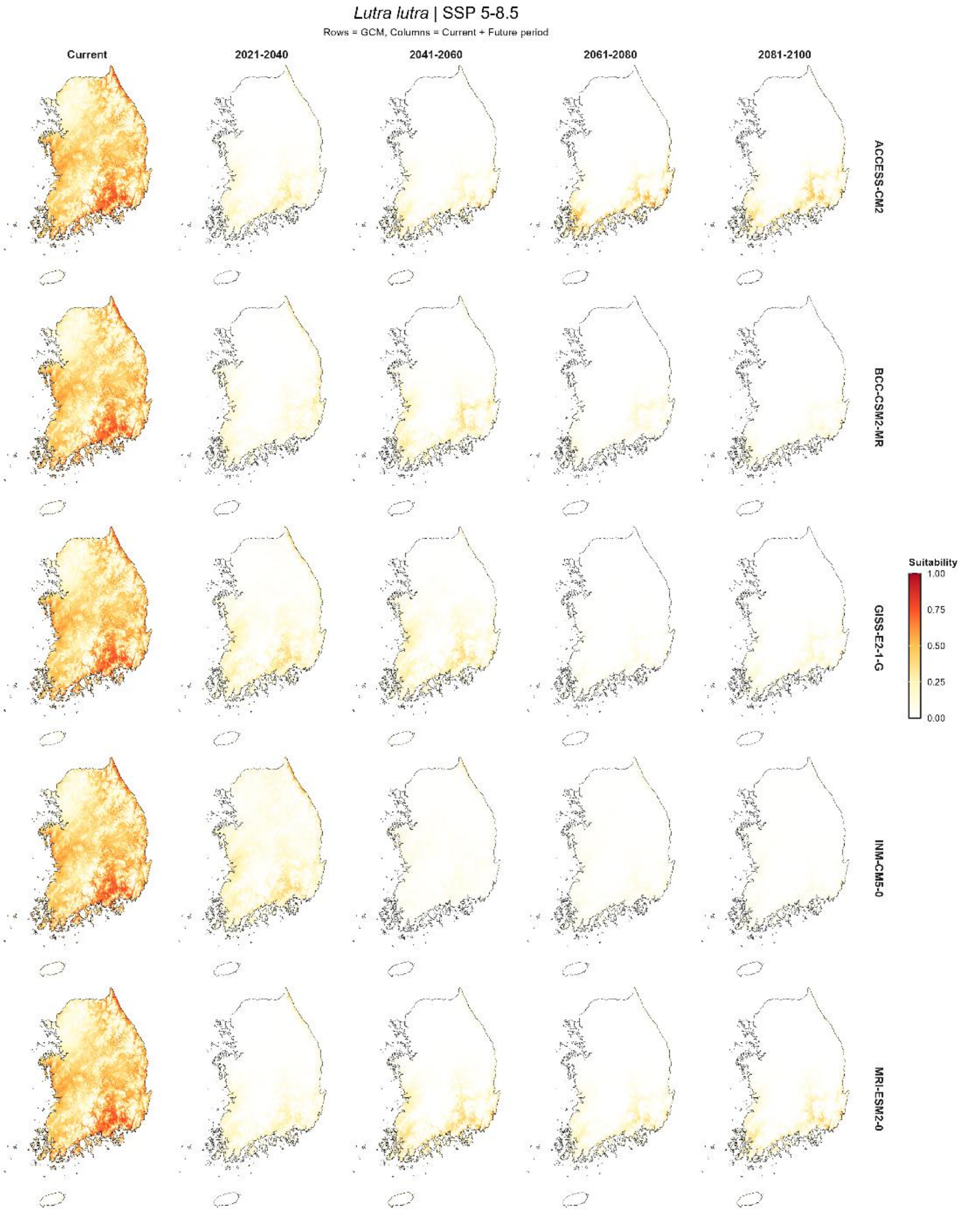
Lutra lutra | SSP 5-8.5
Rows = GCM, Columns = Current + Future period
Current
2021-2040
2041-2060
2061-2080
2081-2100
ACCESS-CM2
BCC-CSM2-MR
GISS-E2-1-G
INM-CM5-0
MRI-ESM2-0
Suitability
1.00
0.75
0.50
0.25
0.00

Martes flavigula

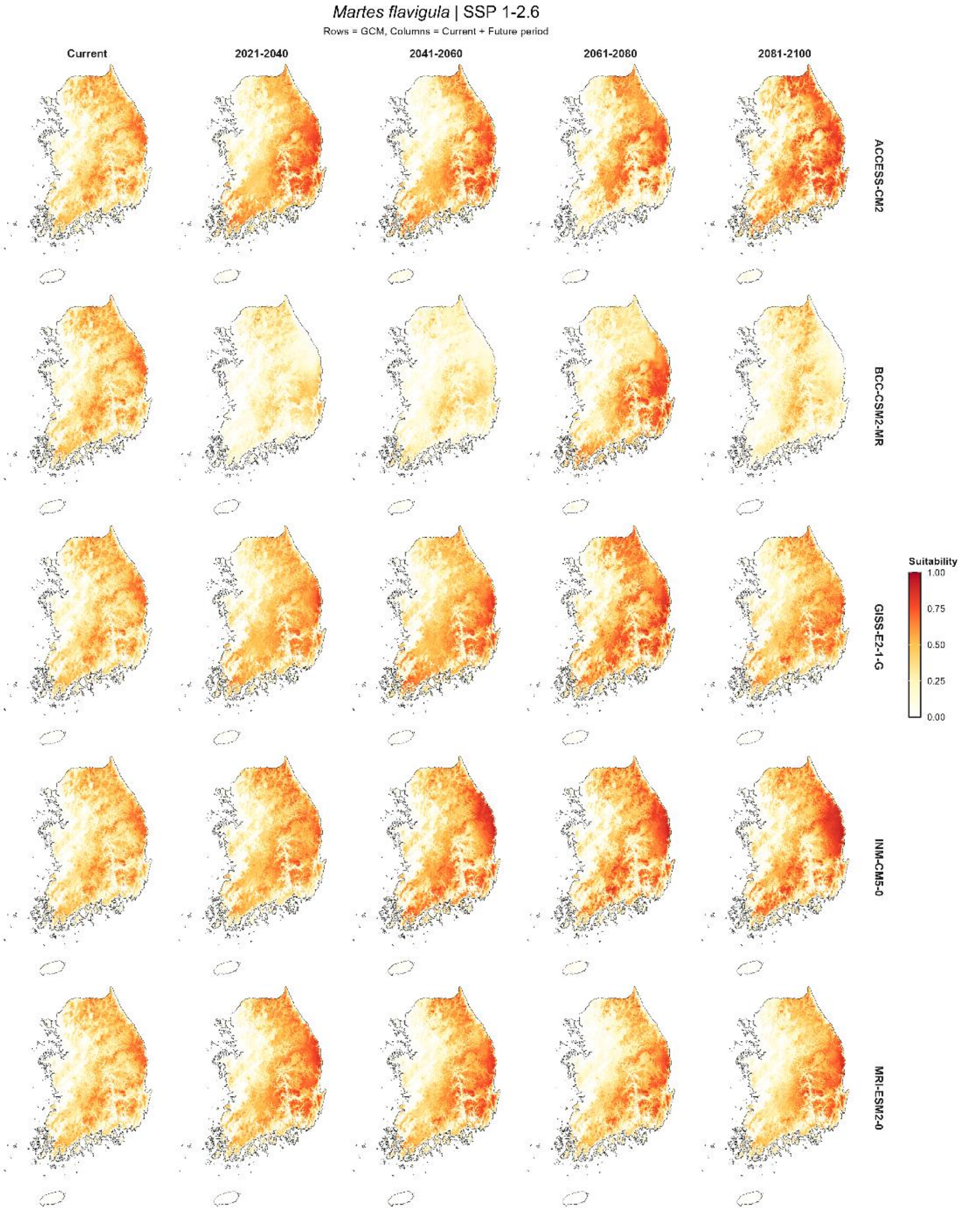

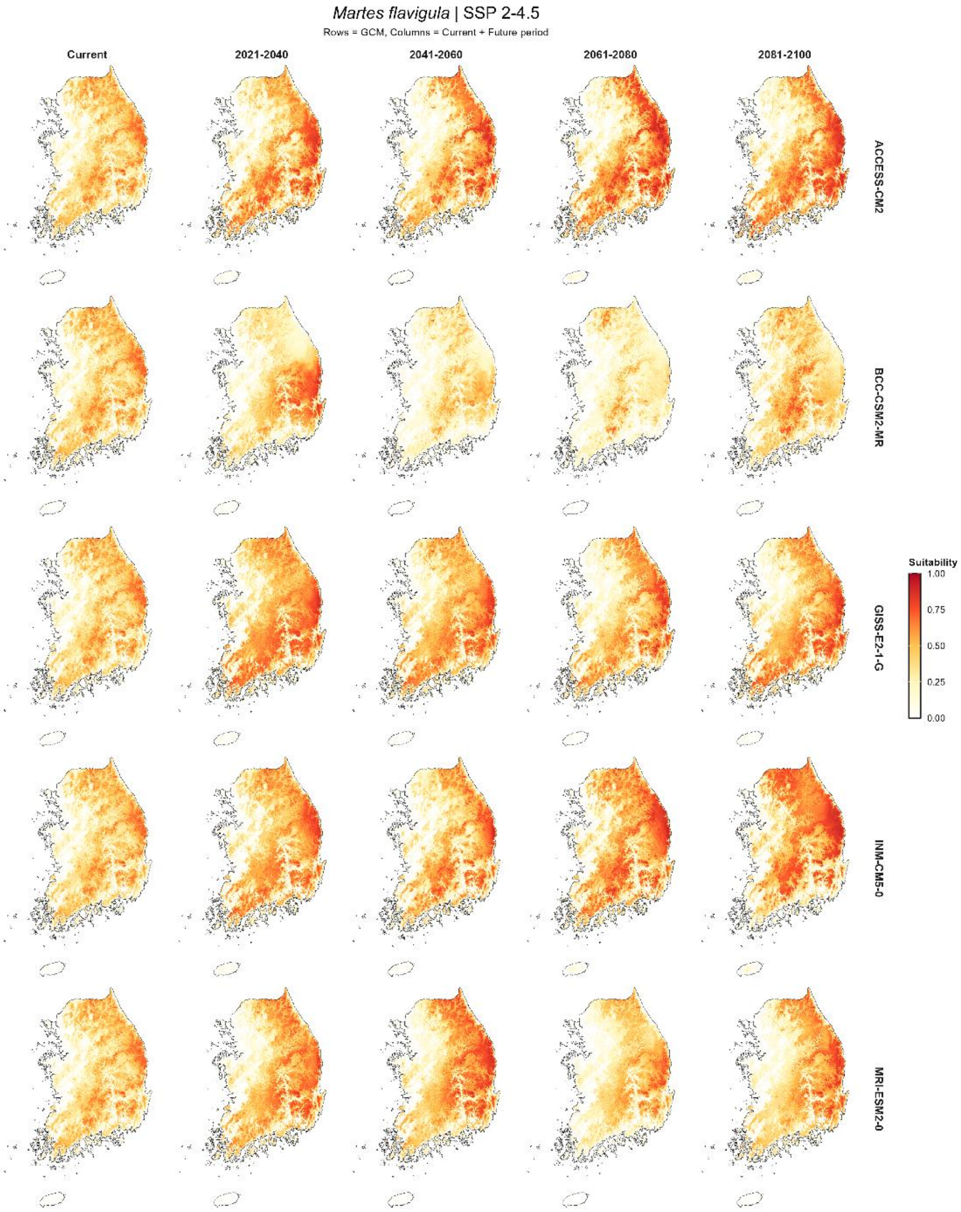

Martes flavigula | SSP 2-4.5
Rows = GCM, Columns = Current + Future period
Current
2021-2040
2041-2060
2061-2080
2081-2100
ACCESS-CM2
BCC-CSM2-MR
GISS-E2-1-G
INM-CM5-0
MRI-ESM2-0
Suitability
1.00
0.75
0.50
0.25
0.00

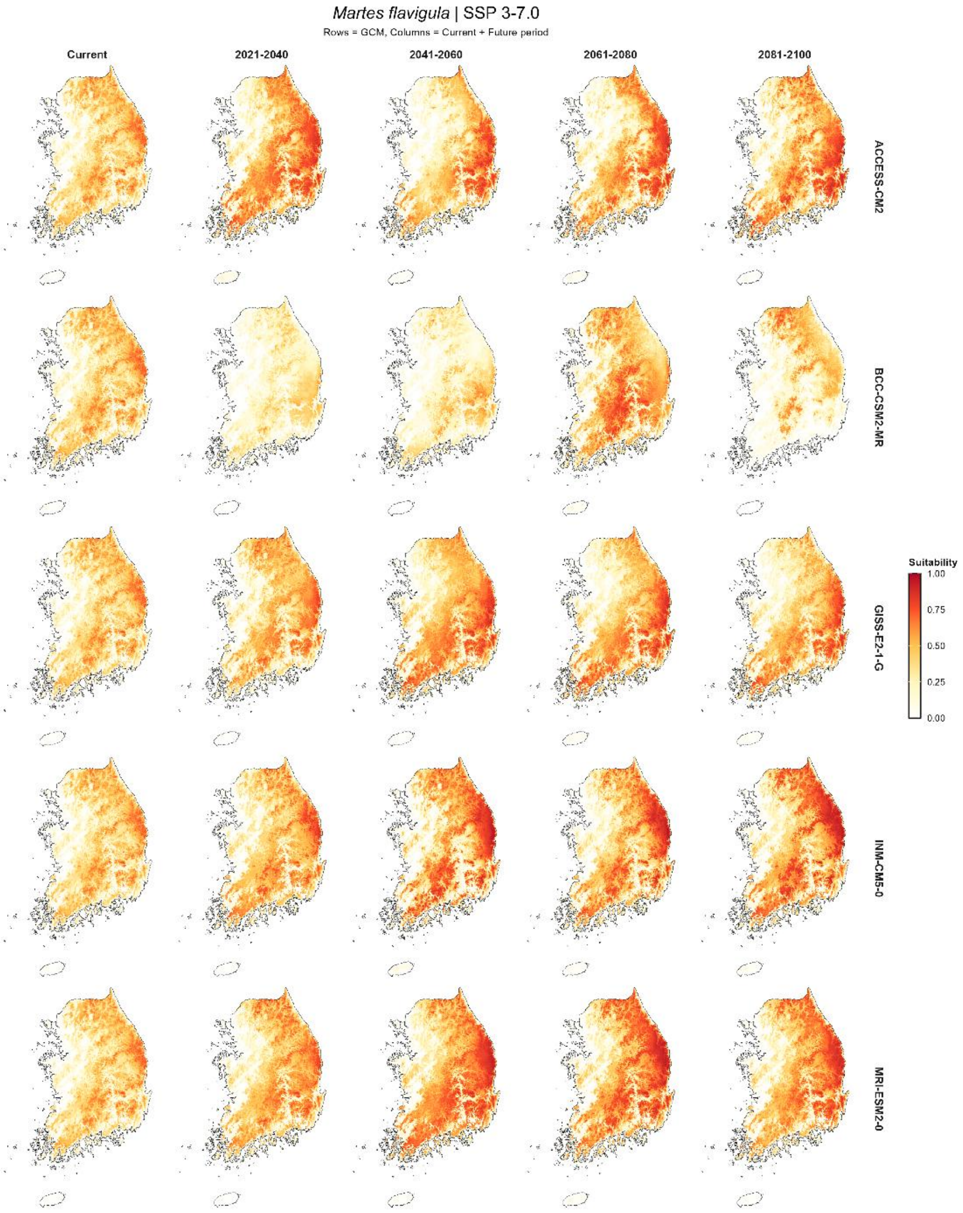
Martes flavigula | SSP 3-7.0
Rows = GCM, Columns = Current + Future period
Current
2021-2040
2041-2060
2061-2080
2081-2100
ACCESS-CM2
BCC-CSM2-MR
GISS-E2-1-G
INM-CM5-0
MRI-ESM2-0
Suitability
1.00
0.75
0.50
0.25
0.00

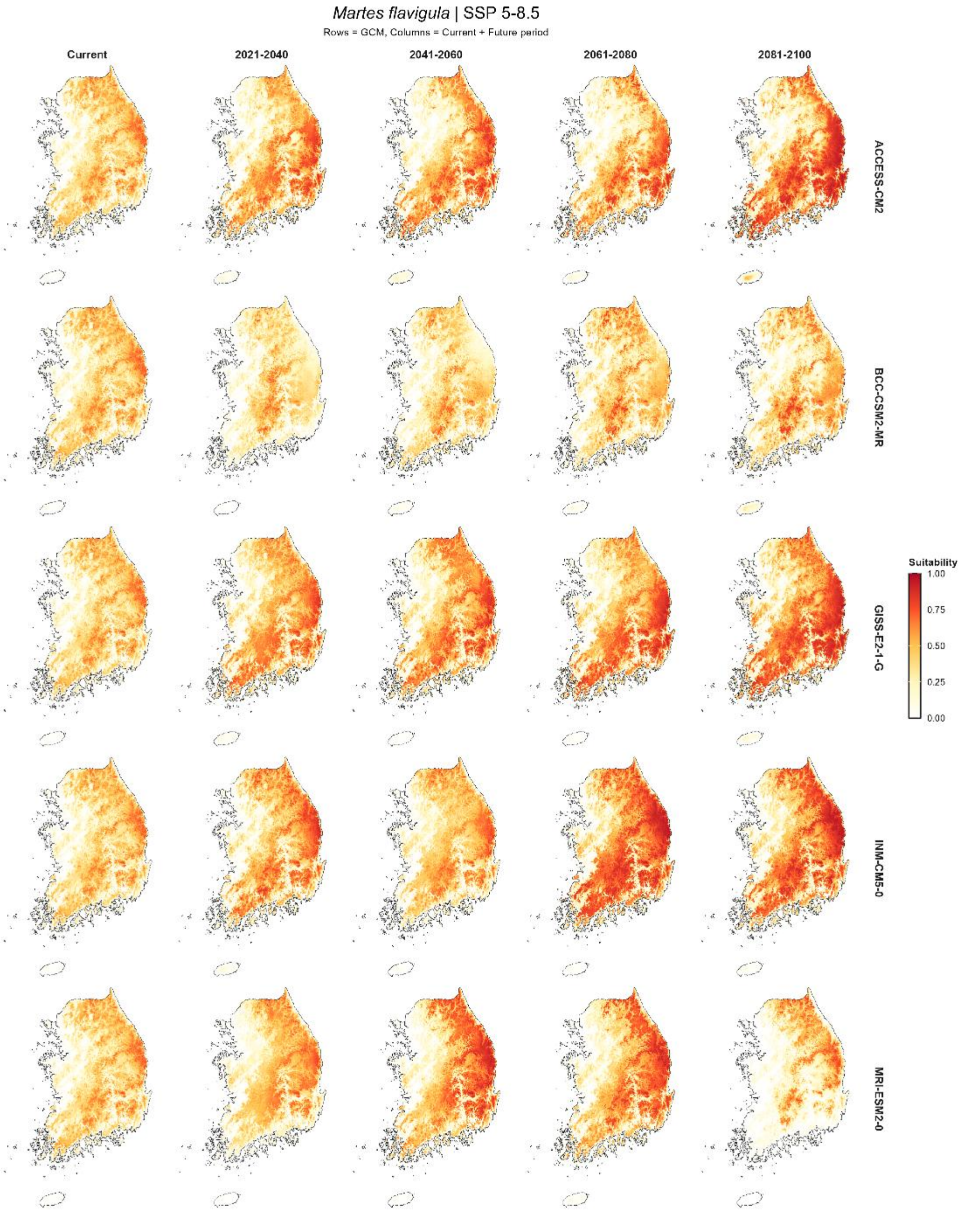
Martes flavigula | SSP 5-8.5
Rows = GCM, Columns = Current + Future period
Current
2021-2040
2041-2060
2061-2080
2081-2100
ACCESS-CM2
BCC-CSM2-MR
GISS-E2-1-G
INM-CM5-0
MRI-ESM2-0
Suitability
1.00
0.75
0.50
0.25
0.00

Naemorhedus caudatus

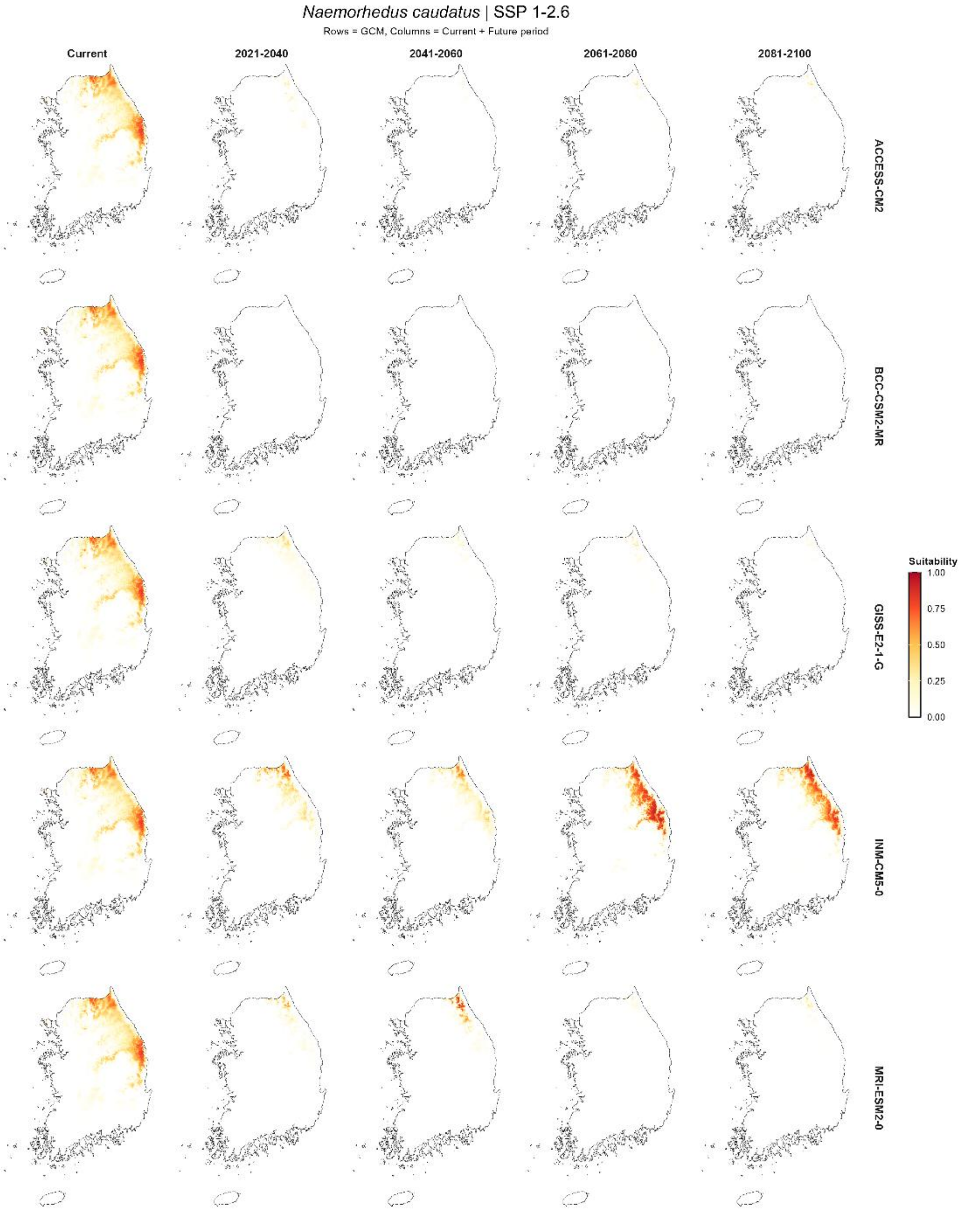

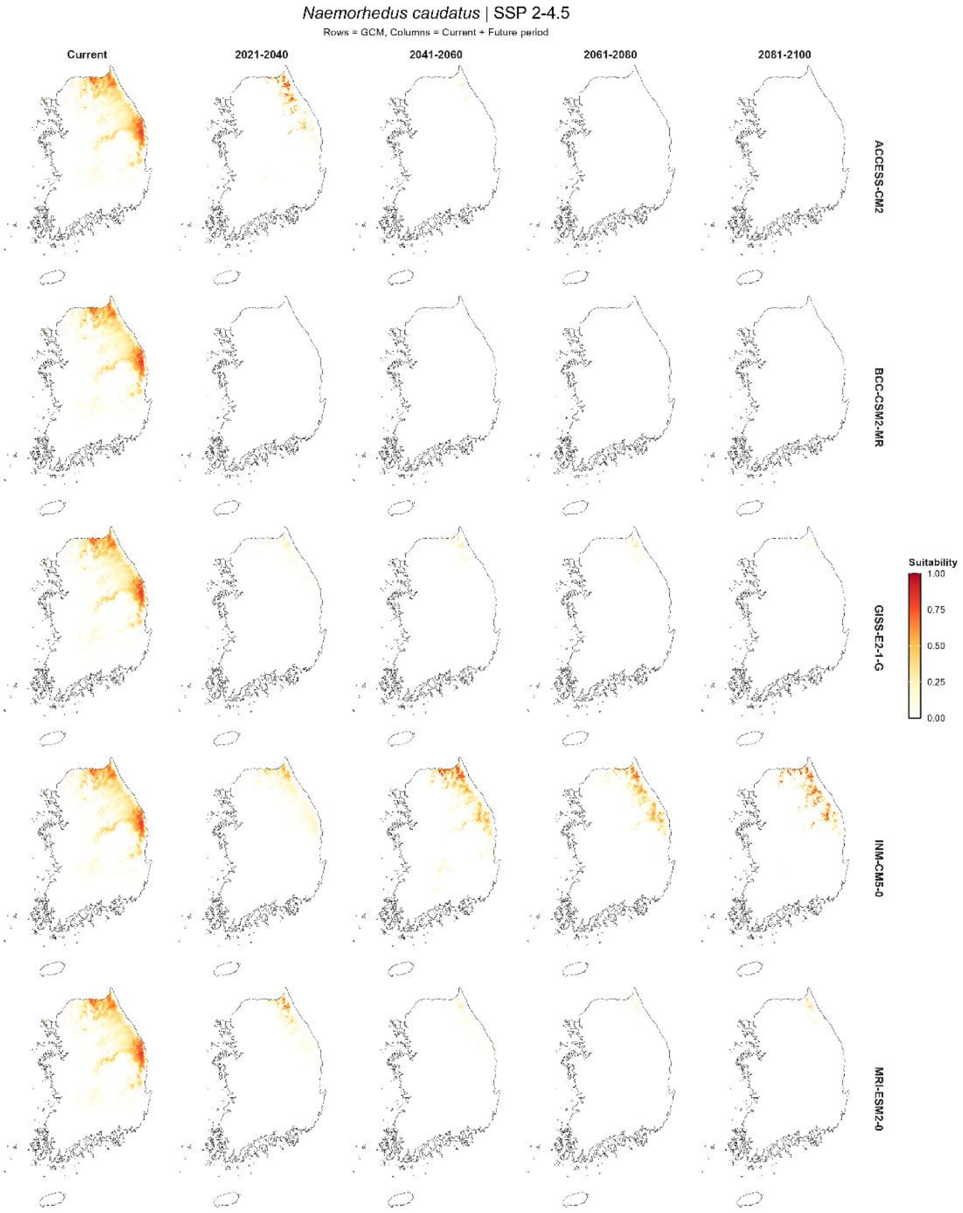
Naemorhedus caudatus | SSP 2-4.5
Rows = GCM, Columns = Current + Future period
Current
2021-2040
2041-2060
2061-2080
2081-2100
ACCESS-CM2
BCC-CSM2-MR
GISS-E2-1-G
INM-CM5-0
MRI-ESM2-0
Suitability
1.00
0.75
0.50
0.25
0.00

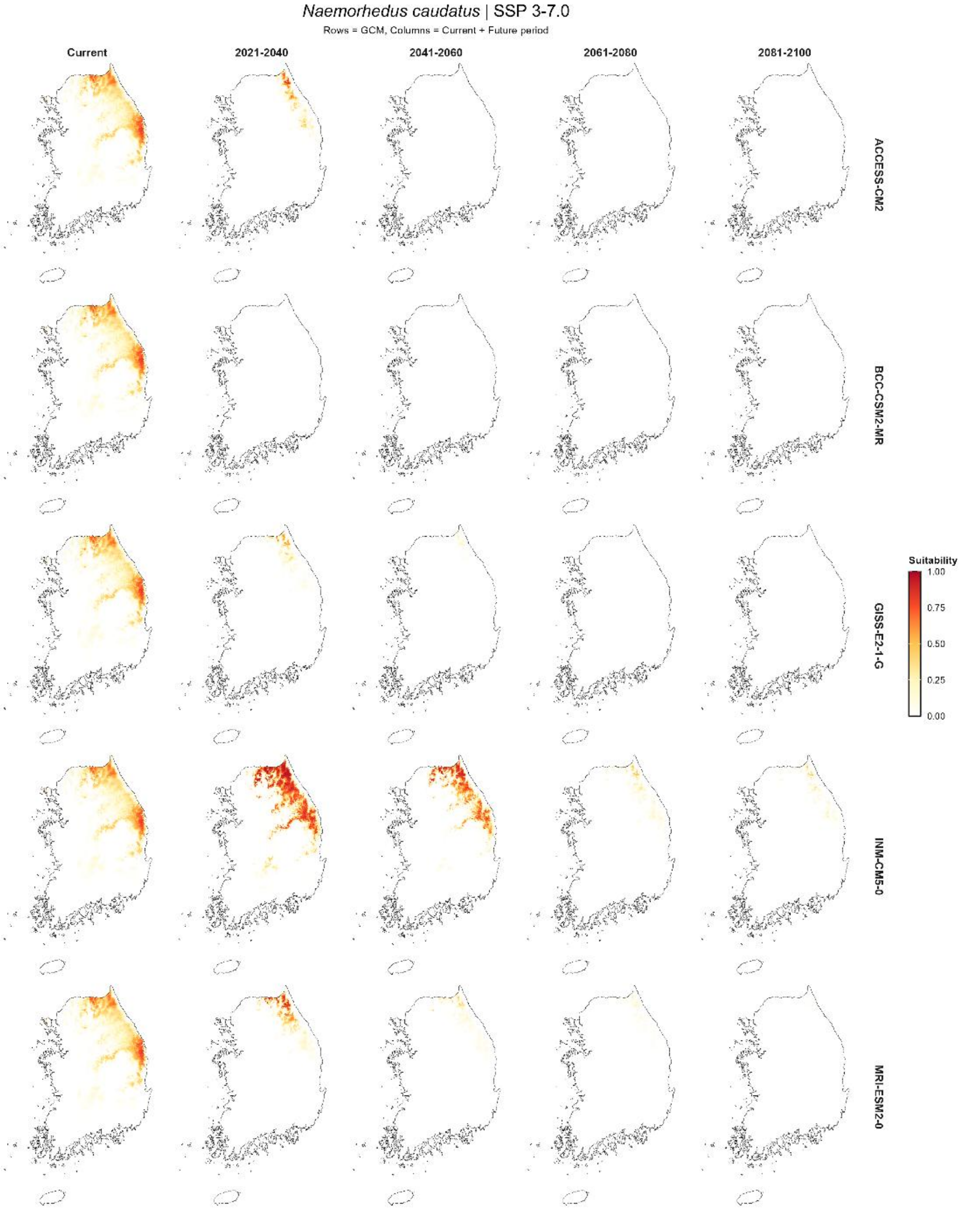

Naemorhedus caudatus | SSP 3-7.0
Rows = GCM, Columns = Current + Future period
Current
2021-2040
2041-2060
2061-2080
2081-2100
ACCESS-CM2
BCC-CSM2-MR
GISS-E2-1-G
INM-CM5-0
MRI-ESM2-0
Suitability
1.00
0.75
0.50
0.25
0.00

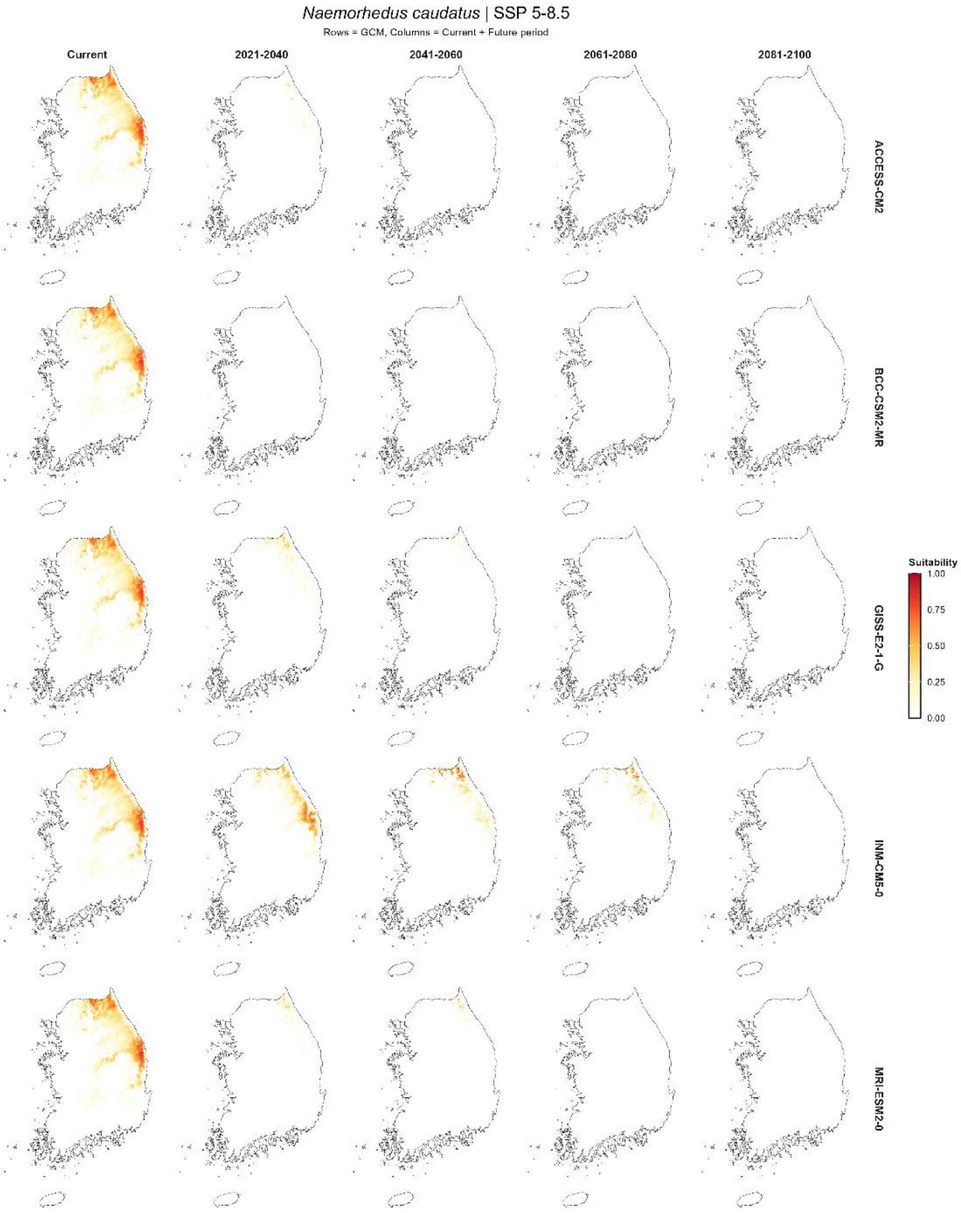

Naemorhedus caudatus | SSP 5-8.5
Rows = GCM, Columns = Current + Future period
Current
2021-2040
2041-2060
2061-2080
2081-2100
ACCESS-CM2
BCC-CSM2-MR
GISS-E2-1-G
INM-CM5-0
MRI-ESM2-0
Suitability
1.00
0.75
0.50
0.25
0.00

Prionailurus bengalensis

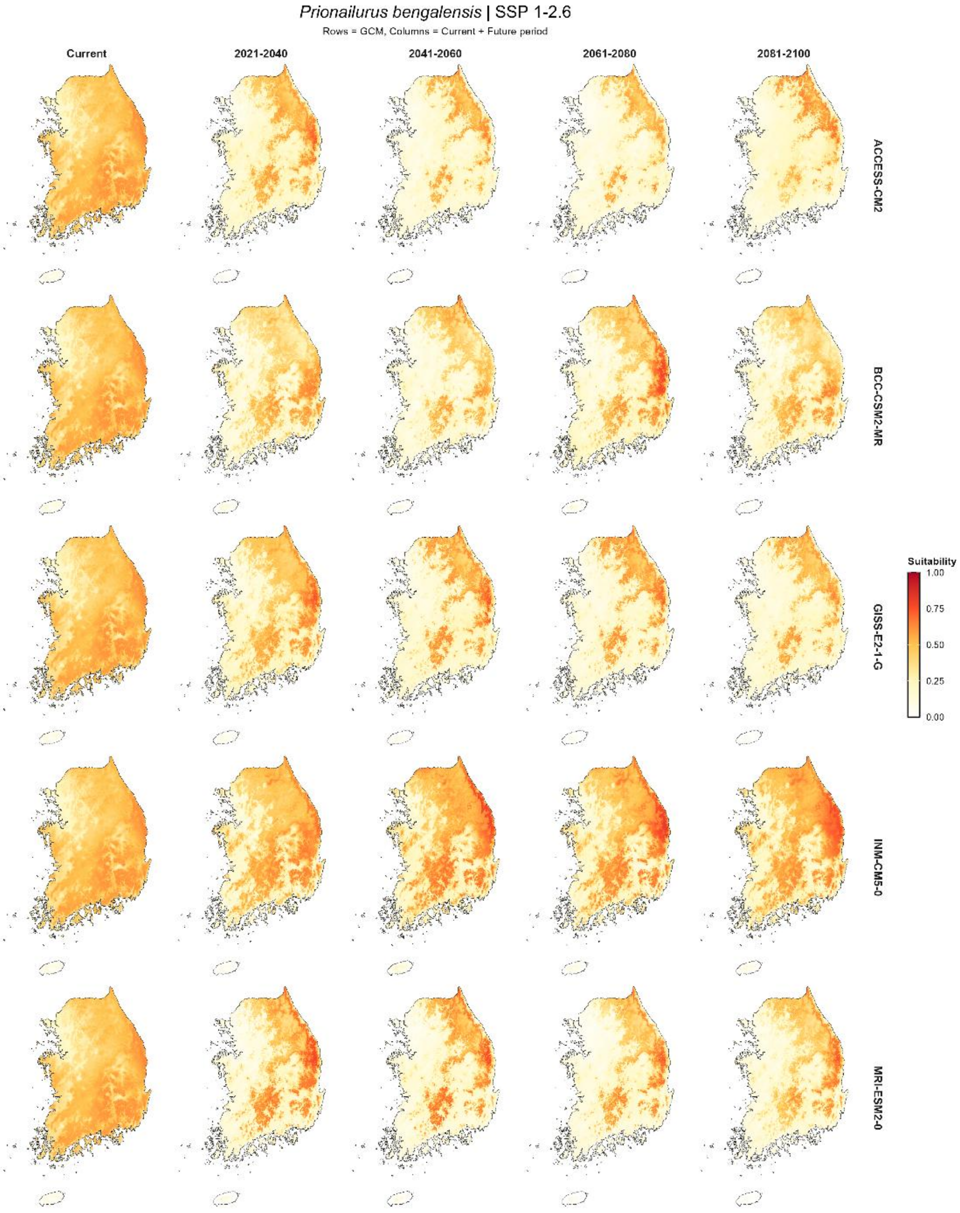

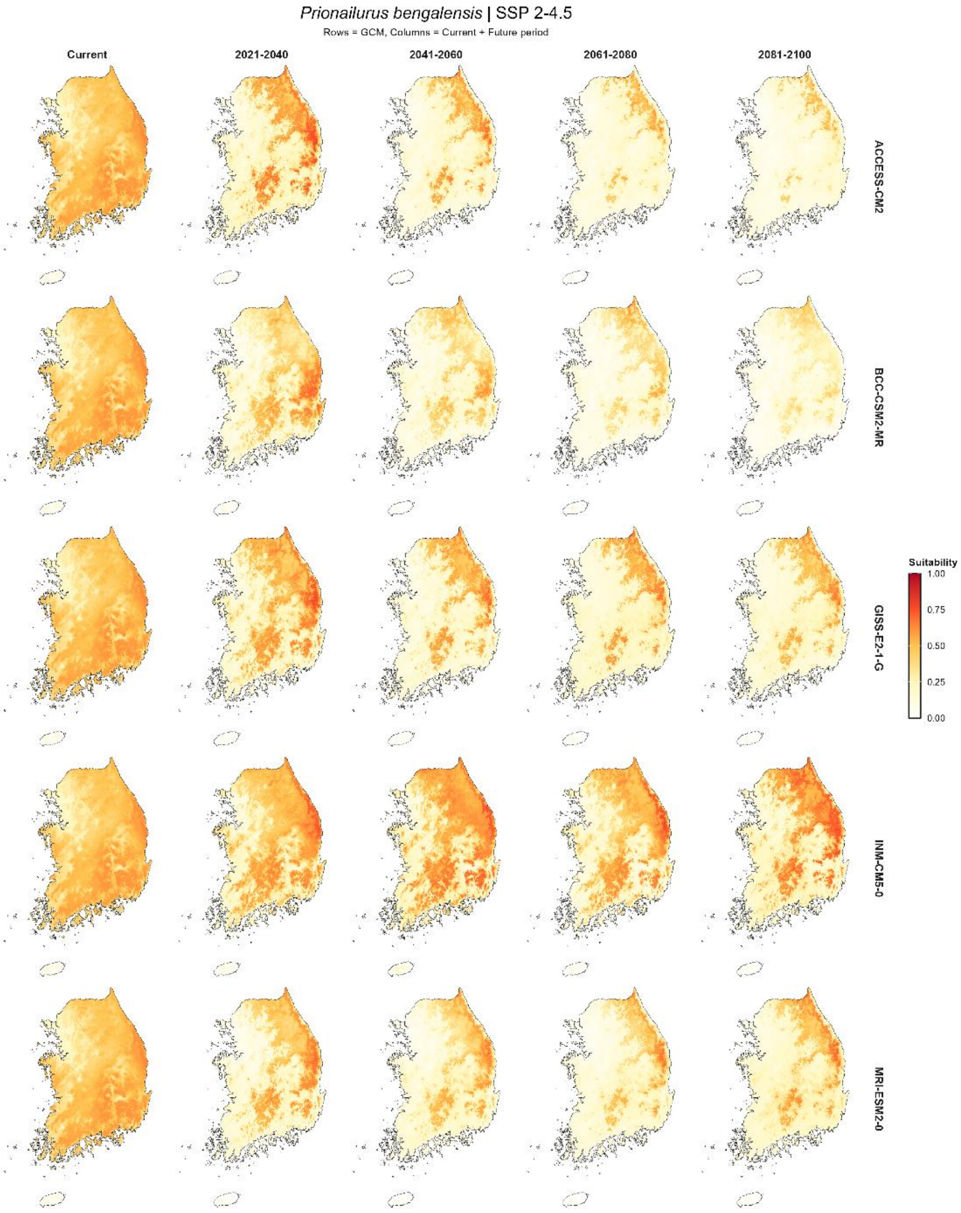

Prionailurus bengalensis | SSP 2-4.5
Rows = GCM, Columns = Current + Future period
Current
2021-2040
2041-2060
2061-2080
2081-2100
ACCESS-CM2
BCC-CSM2-MR
GISS-E2-1-G
INM-CM5-0
MRI-ESM2-0
Suitability
1.00
0.75
0.50
0.25
0.00

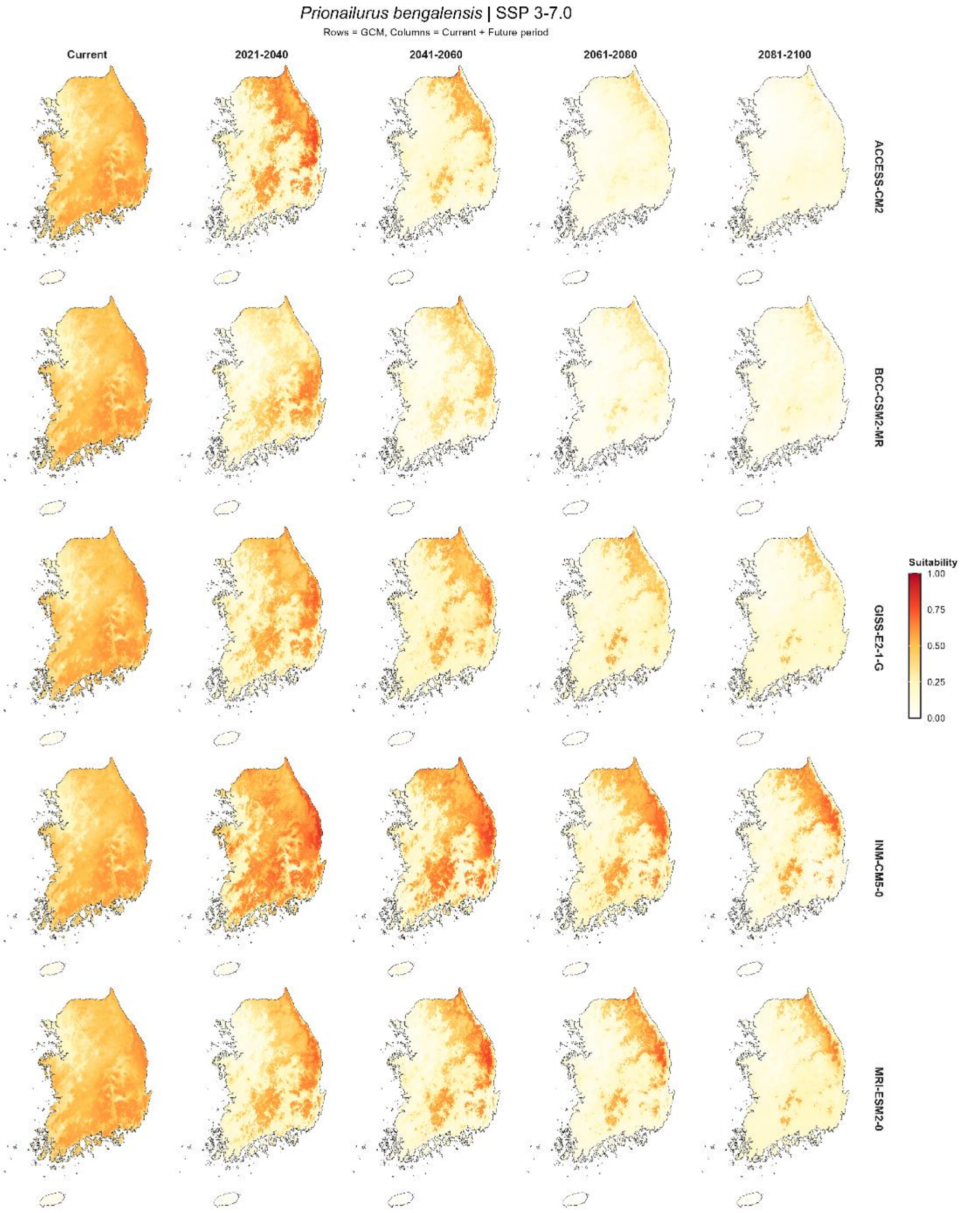

Prionailurus bengalensis | SSP 3-7.0
Rows = GCM, Columns = Current + Future period
Current
2021-2040
2041-2060
2061-2080
2081-2100
ACCESS-CM2
BCC-CSM2-MR
GISS-E2-1-G
INM-CM5-0
MRI-ESM2-0
Suitability
1.00
0.75
0.50
0.25
0.00

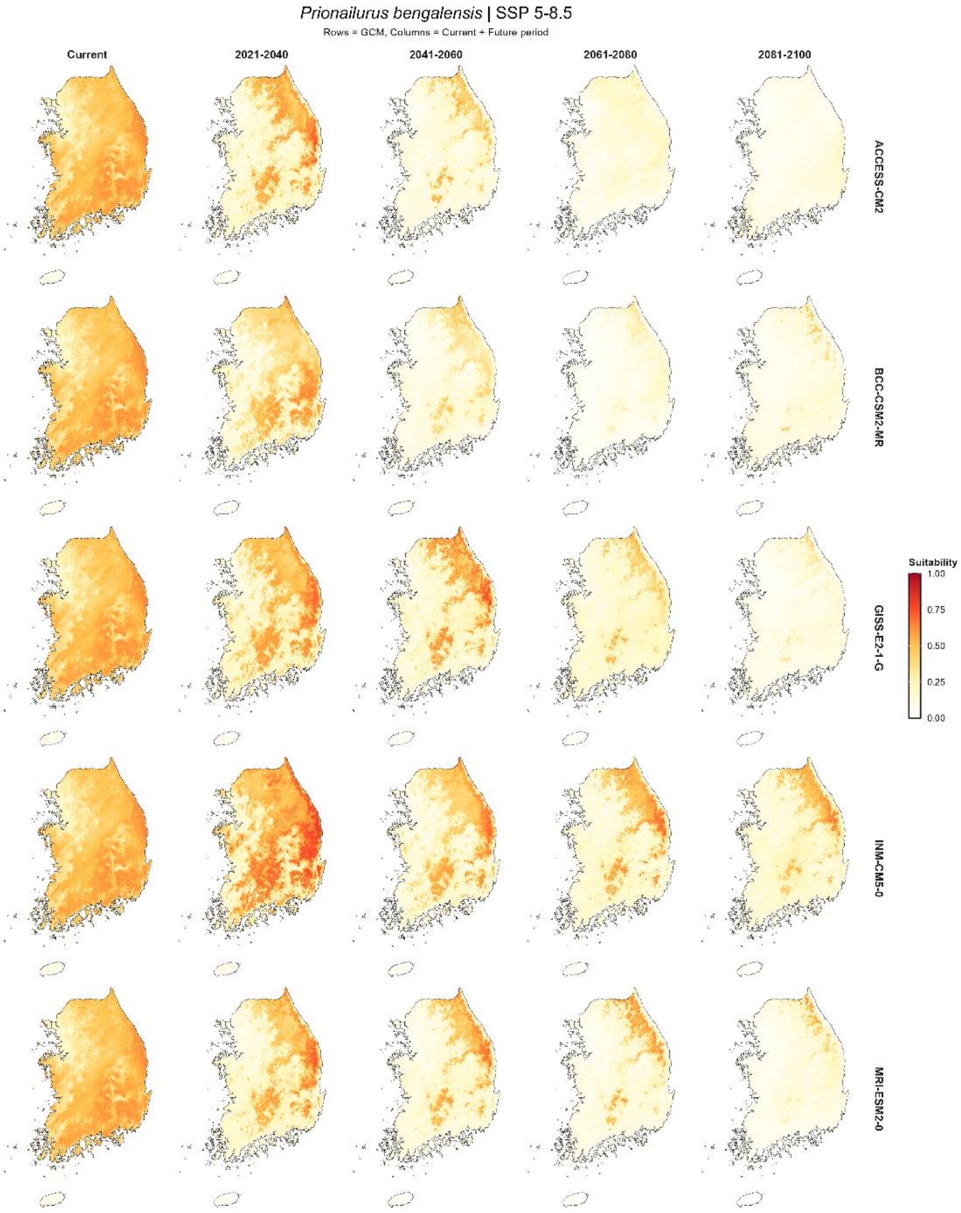

Prionailurus bengalensis | SSP 5-8.5
Rows = GCM, Columns = Current + Future period
Current
2021-2040
2041-2060
2061-2080
2081-2100
ACCESS-CM2
BCC-CSM2-MR
GISS-E2-1-G
INM-CM5-0
MRI-ESM2-0
Suitability
1.00
0.75
0.50
0.25
0.00

Pteromys volans

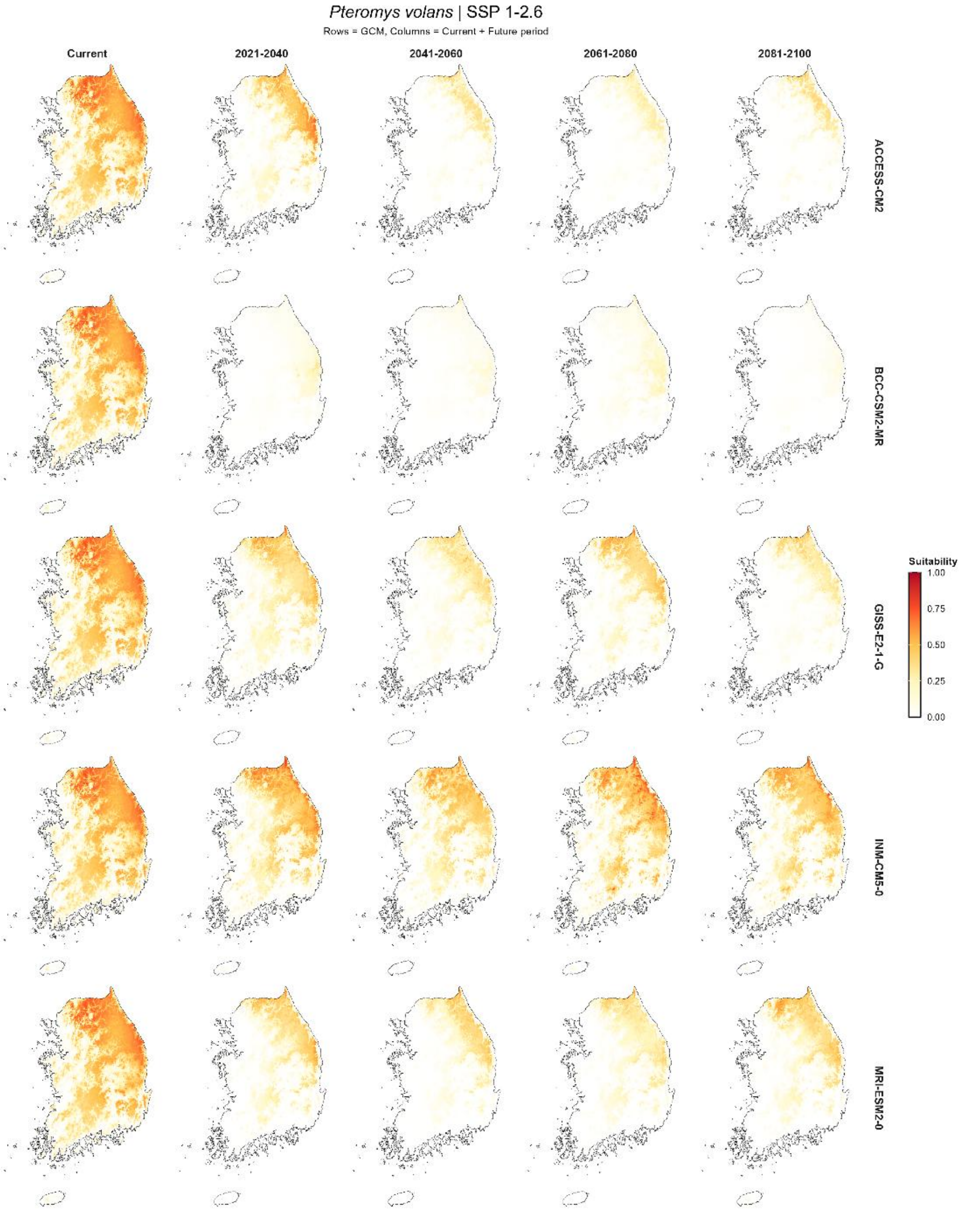

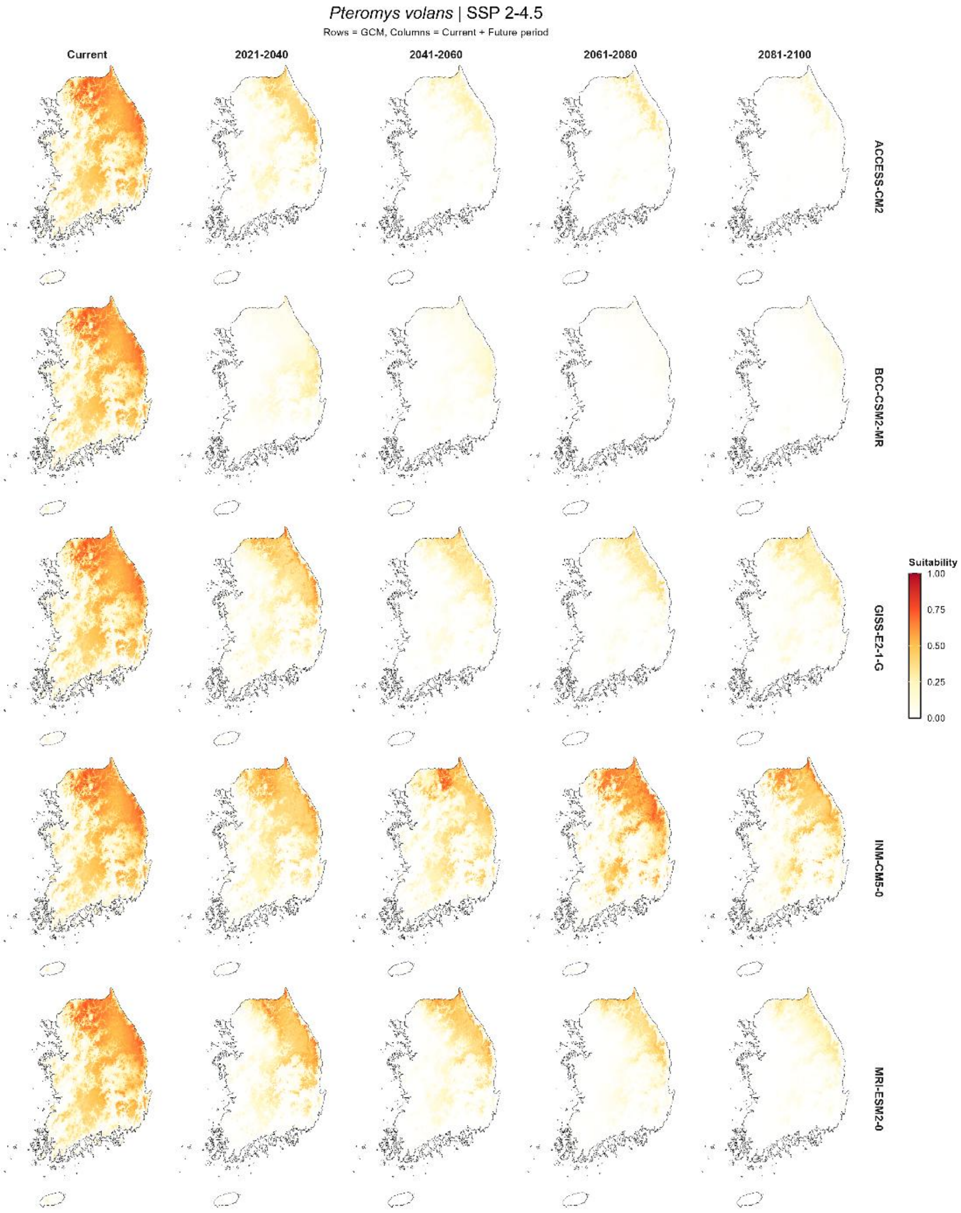
Pteromys volans | SSP 2-4.5
Rows = GCM, Columns = Current + Future period
Current
2021-2040
2041-2060
2061-2080
2081-2100
ACCESS-CM2
BCC-CSM2-MR
GISS-E2-1-G
INM-CM5-0
MRI-ESM2-0
Suitability
1.00
0.75
0.50
0.25
0.00

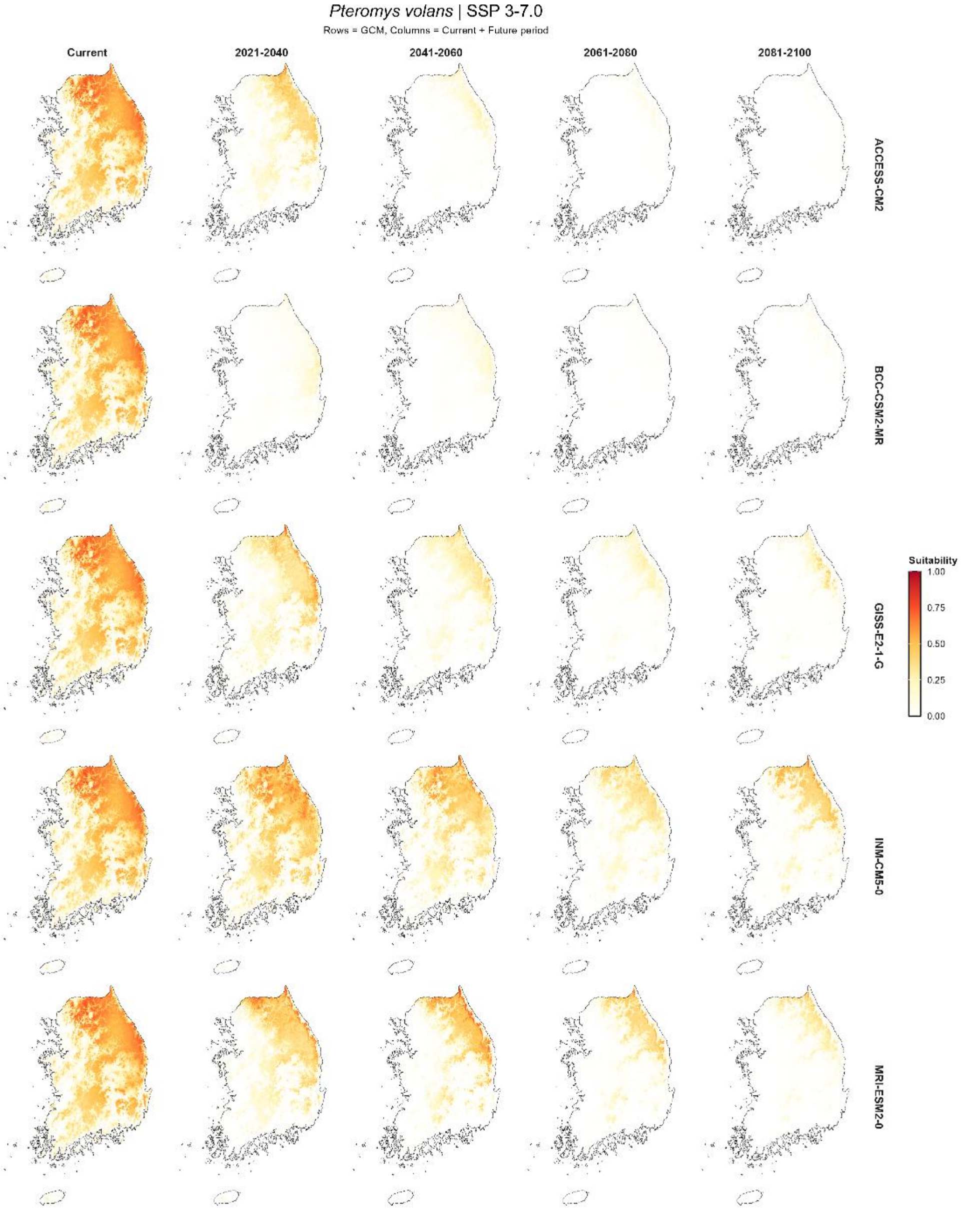

Pteromys volans | SSP 3-7.0
Rows = GCM, Columns = Current + Future period
Current
2021-2040
2041-2060
2061-2080
2081-2100
ACCESS-CM2
BCC-CSM2-MR
GISS-E2-1-G
INM-CM5-0
MRI-ESM2-0
Suitability
1.00
0.75
0.50
0.25
0.00

Pteromys volans | SSP 5-8.5

Rows = GCM, Columns = Current + Future period

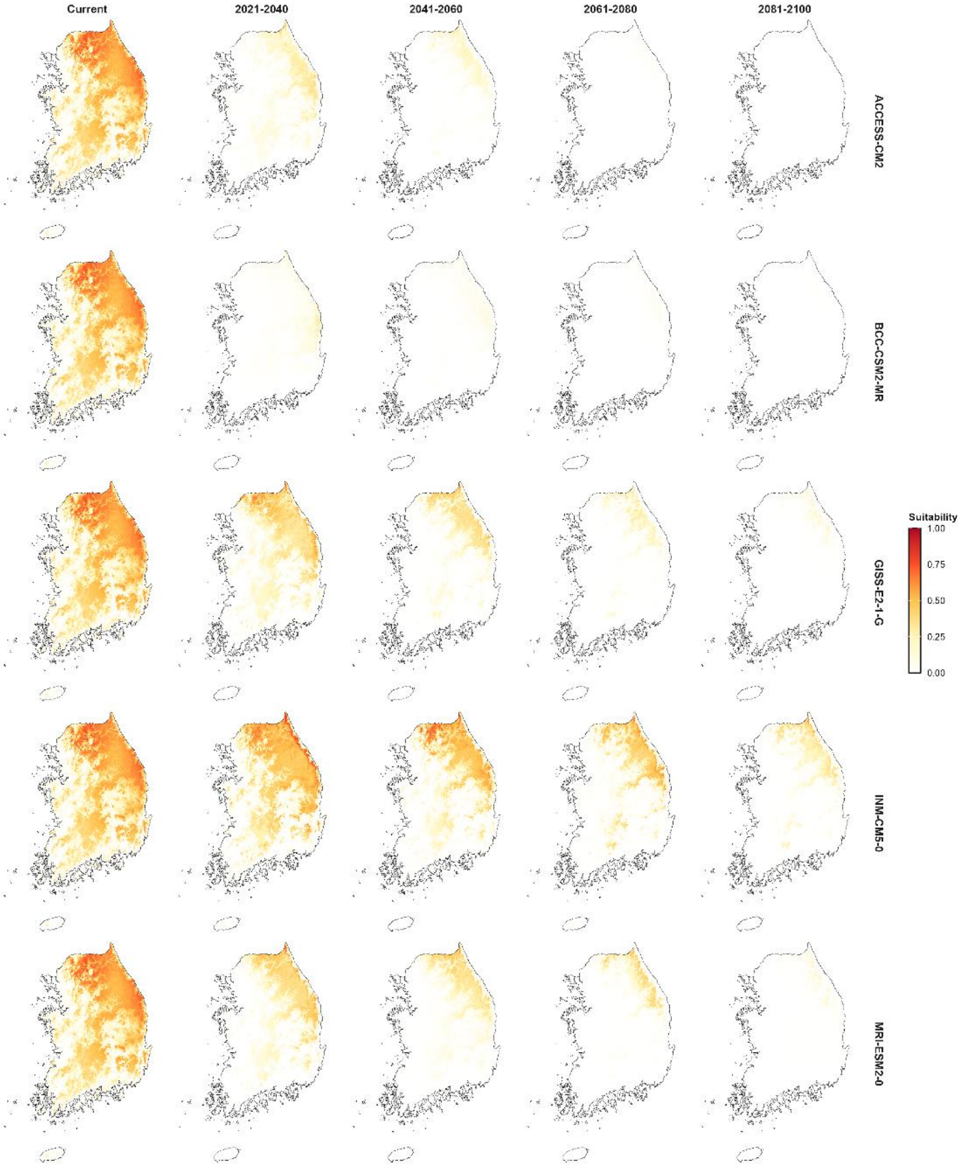

Supplement: Supplementary file 1 [file animals-16-02489-s001.zip › Figure S1 Comprehensive Habitat Suitability Maps for Five Endangered Terrestrial Mammals in South Korea under CMIP6 Climate Change Scenarios.pdf]
